# *Aedes aegypti* post-emergence transcriptome: Unveiling the molecular basis for the hematophagic and gonotrophic capacitation

**DOI:** 10.1371/journal.pntd.0008915

**Published:** 2021-01-06

**Authors:** Stephanie S. de Carvalho, Cynara M. Rodovalho, Alessandro Gaviraghi, Maria Beatriz S. Mota, Willy Jablonka, Carlúcio Rocha-Santos, Rodrigo D. Nunes, Thayane da Encarnação Sá-Guimarães, Daniele S. Oliveira, Ana C. A. Melo, Monica F. Moreira, Patrícia Fampa, Marcus F. Oliveira, Mario Alberto C. da Silva-Neto, Rafael D. Mesquita, Georgia C. Atella

**Affiliations:** 1 Instituto de Bioquímica Médica Leopoldo de Meis, Universidade Federal do Rio de Janeiro, Rio de Janeiro, Rio de Janeiro, Brazil; 2 Departamento de Bioquímica, Instituto de Química, Universidade Federal do Rio de Janeiro, Rio de Janeiro, Rio de Janeiro, Brazil; 3 Laboratório de Fisiologia e Controle de Artrópodes Vetores, Instituto Oswaldo Cruz, Fiocruz, Rio de Janeiro, Rio de Janeiro, Brazil; 4 Instituto Nacional de Ciência e Tecnologia em Entomologia Molecular, Universidade Federal do Rio de Janeiro, Rio de Janeiro, Rio de Janeiro, Brazil; 5 Departamento de Ciências Farmacêuticas, Instituto de Ciências Biológicas e da Saúde, Universidade Federal Rural do Rio de Janeiro, Seropedica, Rio de Janeiro, Brazil; National Institute of Allergy and Infectious Diseases, UNITED STATES

## Abstract

The adult females of *Aedes aegypti* mosquitoes are facultative hematophagous insects but they are unable to feed on blood right after pupae emergence. The maturation process that takes place during the first post-emergence days, hereafter named hematophagic and gonotrophic capacitation, comprises a set of molecular and physiological changes that prepare the females for the first gonotrophic cycle. Notwithstanding, the molecular bases underlying mosquito hematophagic and gonotrophic capacitation remain obscure. Here, we investigated the molecular and biochemical changes in adult *Ae*. *aegypti* along the first four days post-emergence, prior to a blood meal. We performed a RNA-Seq analysis of the head and body, comparing male and female gene expression time courses. A total of 811 and 203 genes were differentially expressed, respectively in the body and head, and both body parts showed early, mid, and late female-specific expression profiles. Female-specific up-regulation of genes involved in muscle development and the oxidative phosphorylation pathway were remarkable features observed in the head. Functional assessment of mitochondrial oxygen consumption in heads showed a gradual increase in respiratory capacity and ATP-linked respiration as a consequence of induced mitochondrial biogenesis and content over time. This pattern strongly suggests that boosting oxidative phosphorylation in heads is a required step towards blood sucking habit. Several salivary gland genes, proteases, and genes involved in DNA replication and repair, ribosome biogenesis, and juvenile hormone signaling were up-regulated specifically in the female body, which may reflect the gonotrophic capacitation. This comprehensive description of molecular and biochemical mechanisms of the hematophagic and gonotrophic capacitation in mosquitoes unravels potentially new targets for vector control.

## Author summary

*Aedes aegypti* is a vector of several human diseases such as Dengue, Zika, Chikungunya, and Yellow Fever. Only the adult females can feed on blood, important for the production of their offspring, and therefore transmitting these pathogens for the human host. Interestingly, right after their emergence as adults, females show a preference of feeding on nectar, and only after three days, they are fully capacitated to perform a blood meal. Understanding the molecular mechanisms that orchestrate this physiological and biochemical switch is the main topic of the present study. The transcriptome analysis of males and females during the first hours post-emergence reveals that the gene expression in females is tightly regulated, showing three time-dependent profiles of expression. There is an increase in genes related to the oxidative phosphorylation pathway in the female head. Assessment of mitochondrial function revealed a gradual increase in respiration linked to ATP synthesis and mitochondrial content. Also, there is an increase in genes related to the muscular function, suggesting a putative role in muscle development and contraction, required, for example, for pumping of blood. The female body showed an increase in salivary gland related genes and serine proteases, probably related to the ingestion and digestion of the blood. Also, the increase in DNA replication, ribosome biogenesis, and juvenile hormone signaling pathways together with changes in cell cycle control suggest the gonotrophic capacitation for the massive synthesis of yolk proteins precursors during vitellogenesis. All these genes were enhanced even before the first blood meal, which reveals insightful information about the biology of anautogenous mosquitoes. Our data showed the molecular machinery that stands the females hematophagy and gonotrophy, leading to potential new targets for vector control.

## Introduction

Mosquitoes are vectors of several human diseases since these insects possess a remarkable human feeding preference [1]. The disease transmission occurs during the blood meal, required by anautogenous female mosquitoes for egg development [2]. *Aedes aegypti* females have a unique dietary skill interchanging between sugary fluids from plants and blood along the gonotrophic cycle. This cycle was named by Beklemishev in 1940 and comprises the host seeking, blood feeding, egg development, and oviposition [3].

Although the first gonotrophic cycle is unique, as the mosquito has never fed on blood before, in the first hours post-emergence (PE) they are unable to feed on blood, acquiring nutrients essentially from plant sap [4]. To get the first blood meal, females must undergo a maturation process in the first three days PE that comprises a series of physiological and molecular adaptations that take place in many tissues [5], named here hematophagic and gonotrophic capacitation (HGC). Juvenile hormone III (JH) is a master regulator PE development, participating in the maturation of newly emerged females towards blood feeding and vitellogenesis [6]. JH acts through its intracellular receptor Methoprene-tolerant (Met), a basic Helix-Loop-Helix/Per-Arnt-Sim (bHLH/PAS) transcription factor [7]. Met forms a heterodimer with Taiman, another bHLH-PAS protein, promoting the transcriptional regulation of several genes [8–10]. The fat body is extremely affected by JH signaling, increasing the expression of ribosomal proteins and driving ribosome biogenesis, nucleolus enlargement, Golgi complex development, and invaginations of the plasma membrane, priming for vitellogenesis [11,12]. JH also induces the immature primary follicles [13,14] to grow twice in length to mature follicles in 48-72h PE and the development of an endocytic complex by the oocytes given them the competence to acquire proteins [13,14].

The JH role on the female fat body was characterized by a post-emergence (PE) microarray in wild-type and Met-depleted females [15]. Three major profiles of expression were observed in the fat body of *Ae*. *aegypti* and in *Culex quinquefasciatus* adult females with an early, mid, and late profiles of transcript abundance [16,17]. The absence of Met revealed a similar profile of gene expression as JH deprived females for a subset of genes. Thus, JH regulates gene expression for several genes through Met during PE development [15]. In a similar approach, the carbohydrate and lipid metabolisms were assessed, through microarray analysis, in the female fat body in order to understand the energy supply for the physiological changes that occur during the PE phase. JH promotes an accumulation of glycogen in the fat body and the transcript abundance of pathways related to carbohydrate metabolism, such as glycolysis and citric acid cycle were high until 24h PE, declining from 36h until 72h PE. Met silencing promotes an increase in carbohydrate metabolism transcripts [18]. Met is also a key regulator of lipid metabolism during the PE hours leading to an accumulation of triacylglycerol (TAG) in the fat body and a reduction in the ß-oxidation and lipid catabolism [12]. Thus, JH plays a role in regulating both carbohydrate and lipid metabolisms during PE development on *Ae*. *aegypti* female mosquitoes.

Some other authors explored the female PE period. The *Culex quinquefasciatus* transcriptome evaluated immediately after the emergence in whole adult females, identified odorant binding proteins, salivary proteins, trypsins, lipases, uricases, among others [17]. The proteome of newly emerged females midgut revealed proteins involved in the metabolism of proteins and amino acids, serine proteases, and cell signaling, suggesting that there is a biochemical adaptation for the digestion of blood in the PE developmental phase [19]. The salivary glands transcriptome compared female to male, in a single time-point PE, revealing sex-specific proteins [20]. Tallon *et al* assessed the expression of main chemosensory gene families comparing female to male antennae, in a post-emergence time course using a transcriptome. Odorant binding proteins, odorant receptors, and ionotropic receptors gene expression were sex-specific and time-dependent [21]

Given the inability to feed on blood right after emergence, and the voracious hematophagous habit on mature stages, an important gap of knowledge exists on the molecular and biochemical mechanisms that mediate hematophagic capacitation in mosquitoes. The HGC occurs in the PE phase before the first blood feeding and are female-specific features, so only the comparison of female and male time-course transcriptomes will target the genes related to them. In the present work, we used RNA-Seq to investigate the effect of sex and time on the dynamics of gene expression in the first 96 hours PE, during HGC. The separated analysis of heads and bodies provided a global view of the complex and tightly regulated molecular remodeling that the females undergo along the first post-emergence hours. The transcriptome analysis revealed sex- and time-dependent changes in transcript abundance, with 203 genes differentially expressed in the head and 811 in the body. The up-regulation of the oxidative phosphorylation pathway in female heads lead us to perform a functional assessment of mitochondrial function in mosquitoes heads, revealing an increase in respiration linked to ATP synthesis and mitochondrial content. We discuss our findings in relation to the current knowledge of the physiological features concerning blood feeding behavior and the gonotrophic cycle in mosquitoes.

## Methods

### Ethics statement

All animal care and experimental protocols were conducted following the guidelines of the institutional care and use committee (Committee for Evaluation of Animal Use for Research at the Federal University of Rio de Janeiro, CAUAP-UFRJ), Fundação Oswaldo Cruz Animal Use Ethics Committee (CEUA Fiocruz), and the NIH Guide for the Care and Use of Laboratory Animals (ISBN 0-309-05377-3). The protocols were approved by CAUAP-UFRJ under the registry #IBQM067-05/16 and 24154319.5.0000.5257 and CEUA Fiocruz LW-20/14. Technicians dedicated to the animal facility at both places conducted all the aspects related to animal husbandry under strict guidelines to ensure their careful and consistent handling.

### Mosquito rearing

All experiments were performed with a colony previously established by our group designated as *Aedes* Rio [22]. Ovitrap collected eggs from 5 different places around the Guanabara Bay: Paquetá Island, São Gonçalo, Praça XV, Niterói, and Governador Island were hatched, raised into adults, identified as from *Ae*. *aegypti*, and then sorted into males and females. A pool of 20 males from each site was mated with 20 virgin females of every other location, for 5 days. After mating, all females were placed in a cage for blood feeding and egg laying (*Aedes* Rio F1 generation). The *Aedes* Rio F1 generation mating was performed with 500 males and 500 females (1:1). From F2 generation onwards the natural female/male proportion (1:2) was not manipulated. The eggs were hatched in deoxygenated filtered water for 2 h and maintained at the Laboratory of Physiology and Control of Arthropods Vectors (LAFICAVE-Fiocruz), under BOD controlled conditions (27°C ± 1°C, 12:12h light:dark cycle, and 75% ± 10% relative humidity). First instar larvae were pooled in groups of ~300 per tray, in 1 liter of deoxygenated filtered water, and 1 g of cat food (Friskies, Purina) was provided every three days. Pupae were collected only once, on the day in which their number was the highest, and transferred to cardboard cages (17 cm diameter x 18.5 cm high). *Aedes* Rio F4 generation was used in the transcriptome, the details about mosquito collecting and sample preparation are in the topic "Sample preparation and RNA-Seq". For the other experiments, *Aedes* Rio F8 to F10 generation eggs were hatched at the Federal University of Rio de Janeiro and maintained as described above. Adult mosquitoes were collected in a two-hour window, i.e., from 1 hour before until 1 hour after each time point. The adult mosquitoes were maintained with 10% sucrose *ad libitum*. Females and males, heads and bodies, were collected 2, 12, 24, 48, and 96 hours after the emergence from pupae.

### Seeking assay

The human host proximity assay was adapted from [23]. *Aedes* Rio female mosquitoes (F8-F10 generation) were kept with 10% sucrose *ad libitum*, but not blood fed. The post-emergence time points tested were 2, 12, 24, 48, and 96 hours. Fifteen minutes before each assay, 10 adult females were placed in an acrylic cage (16 x 16 x 16 cm) and human volunteers (N = 5, 2 females, aged 20–29) placed their right forearm approximately 2.5 cm away from the cage for 5 minutes. There was no direct contact between mosquitoes and the volunteers. A Sony Cyber-shot camera was positioned right across the cage, allowing a plain view of the mosquitoes and the forearm. This system was isolated with styrofoam at all sides, preventing interference from the environment and blocking not-exposed net areas. For each assay a 10-minute video was recorded, 5 minutes without and 5 minutes with the human forearm. The number of mosquito visits to the net was counted, with and without the forearm, considering only the exposed net area. P<0.005; Kruskal-Wallis corrected one-way ANOVA test comparing the hours post-emergence were performed using GraphPad Prism software v.6.0.

### First feeding assay

This assay was adapted from [17]. *Aedes* Rio mosquitoes (F8-F10 generation) were tested at the following post-emergence time points: 2, 12, 24, 48, and 96 hours. All mosquitoes were kept with 10% sucrose *ad libitum* and sugar-starved for 12h before each blood meal offer, except for the 2-hour post-emergence time point. For each time point, a cage containing approximately 60 mosquitoes of both sexes (~1:2 ratio) was fed in an artificial membrane feeding system with rabbit blood at 37°C for 30 minutes. Mosquitoes were not manipulated from their emergence to the blood meal offer. The number of fed females was evaluated right after each assay. Midguts from fed and unfed mosquitoes were dissected and stored at -70°C in protease inhibitors cocktail until protein quantification by Lowry assay [24] (4 independent experiments were performed). P<0.05; Kruskal-Wallis corrected one-way ANOVA test comparing the hours post-emergence. P<0.0001; Tukey’s corrected one-way ANOVA test comparing the protein midgut contents in blood fed and non-blood fed female mosquitoes in the hours post-emergence. Both tests were performed using GraphPad Prism software v.6.0.

### Sample preparation and RNA-Seq

*Aedes* Rio mosquitoes (F4 generation) were kept with 10% sucrose *ad libitum*, but not blood fed. The post-emergence time points tested were 2, 12, 24, 48, and 96 hours. All samples were collected at the same time each day (8:30 a.m.), except for the 12 hours time point that was collected twice—at day (8:30 a.m.) and at night (8:30 p.m.). Males and females were immobilized by chilling on ice onto a pre-cooled Petri dish and then dissected to obtain the heads and body (Pools of 10, N = 3). The samples were stored in RNAlater (Invitrogen) at -70°C, and then total RNA was extracted with the miRNAeasy Mini Kit (Qiagen) following the manufacturer's instructions. The 72 cDNA libraries were prepared with Truseq RNA Library Preparation (Illumina) and RNA-Seq sequencing was performed in Illumina HiSeq 4000, with 50 bp single-read, by the AgriLife Genomics and Bioinformatics Service (https://www.txgen.tamu.edu/) at Texas A&M University. Raw sequence reads were deposited at SRA under bioproject accession PRJNA659517.

### Bioinformatics analysis

Sequenced reads were analyzed using FastQC version 0.11.5 [25] to evaluate their quality, before and after trimming with Cutadapt version 1.16 [26]. Adaptors and regions with quality scores smaller than 32 were removed, the remaining reads were dropped if the length was below 40 bp. These high-quality reads were aligned using Bowtie2 version 2.2.6 [27] to the *Ae*. *aegypti* transcripts (version 5.1—downloaded from VectorBase https://www.vectorbase.org/downloadinfo/aedes-aegypti-lvpagwgtranscriptsaaegl51fagz). Salmon version 0.9.1 [28,29] was used with standard parameters to quantify the transcripts due to the high amount of isoforms in this *Ae*. *aegypti* transcript version. The R package DESeq2 [30] allowed the identification of differentially expressed genes (DEG). The likelihood ratio test (LRT) (p-value <0.05) was used to time course DEG identification (male x female) while the Wald test (p-value <0,05; LogFoldChange > 1 or 2) followed by a false discovery rate (FDR—p-value <0.05) to compare each time point individually between males and females. Only DEG identified by both methods were considered. Hierarchical clusterization and heatmaps used Expander version 7.1 [31]. Enrichment analysis of the three principal categories (Biological Process, Molecular Function, and Cellular Component) of gene ontology (GO) was performed with Panther Scoring Tool [32]. Statistical support was provided by the FDR corrected Fisher test (p-value <0.1). The *KEGG Mapper–Search&Color Pathway tool*, from the Kyoto Encyclopedia of Genes and Genomes (KEGG) database, was used to highlight the differentially expressed genes in pathways [33–35].

### Quantitative PCR (qPCR) validation

*Aedes* Rio mosquitoes (reared from F9-F10 eggs) were kept with 10% sucrose *ad libitum*, but not blood fed. The post-emergence time points tested were 2, 24, and 96 hours. Mosquitoes were immobilized by chilling on ice and then dissected to obtain the heads and body (two pools of 5 mosquitoes from each of three independent cages). The samples were immediately fixed on TRIzol Reagent (Thermo Fisher Scientific) and the tissues were homogenized with pistils. The samples were stored at -70°C for about 5 days until RNA extraction according to manufacturer instructions. RNA was analyzed through a nanodrop spectrophotometer (Thermo Fisher Scientific) for quality and quantity and 1μg of RNA of each sample was treated with DNAse I (Thermo Fisher Scientific). cDNA was prepared using High Capacity cDNA Reverse Transcription Kit (Thermo Fisher Scientific) according to the manufacturer instructions and the qPCR was performed in a StepOnePlus Real-Time PCR System (Thermo Fisher Scientific). The qPCR reactions were performed with HOT FIREpol EvaGreen qPCR Mix Plus (Solis Biodyne) with a final volume of 10μL, using 2μL of the reagent, 0.1μM of each primer, and 5μL of cDNA (1:50). The primers efficiency was established through calibration curves and determined from slope. The genes for qPCR validation were selected based Li et al (2019) [36]. One gene for each female enhanced cluster was chosen, except B4, H2, and H3 that did not fit the method criteria. The reference genes were selected based on the analysis of the transcriptome expression levels [36]. The Cq values were analyzed in Refinder software that also calculates ΔCq. The less variable genes were eukaryotic translation factor 1A (eiF1A) and eukaryotic translation factor 3, subunit J (eiF3j) that were used as reference genes following MIQE guidelines [37] The primers used for this analysis were provided in **S3 Table**. Expression levels were calculated using 2^-ΔCT^. p-value <0.05; two-way ANOVA test was used to analyze the factors time and sex. Tukey´s (time) and Sidak´s (sex) were used to correct the multiple comparisons. Tests were performed using GraphPad Prism software v.6.0.

### Respirometry analyses on mechanically permeabilized heads

Respiratory activity of mechanically permeabilized heads from *Aedes* Rio mosquitoes was performed using a two-channel titration injection respirometer (Oxygraph-2k, Oroboros Instruments, Innsbruck, Austria) according to a method previously established by our group [38,39]. *Aedes* Rio mosquitoes (reared from F8-F10 eggs) were kept with 10% sucrose *ad libitum*, but not blood fed. The post-emergence time points tested were 2, 12, 24, 48, and 96 hours. Males and females were dissected and a pool from 15 heads was placed into the O2K chamber filled with 2 mL of respiration buffer (120 mM KCl, 5 mM KH_2_PO_4_, 3 mM HEPES, 1 mM EGTA, 1.5 mM MgCl_2_, and 0.2% fatty acid free bovine serum albumin, pH 7.2). The samples were subjected to a mechanical permeabilization inside the respirometer chamber by stirring at 750 rpm for about 10 minutes. The analysis was performed at 27.5°C and 750 rpm. The substrate–uncoupler–inhibitor titration (SUIT) protocol was started by adding 10 mM pyruvate followed by 10 mM proline (Pyr+Pro). This combination of substrates was used because pyruvate and proline represent two of the main substrates used by *Ae*. *aegypti* as was shown previously by our group [40]. Then, 2 mM ADP was added and the oxygen consumption coupled to ATP synthesis (ATP linked respiration) was calculated by subtracting the oxygen consumption after substrate addition from ADP-stimulated oxygen consumption rates. The maximum uncoupled respiration was induced by stepwise titration of carbonyl cyanide p-(trifluoromethoxy) phenylhydrazone (FCCP) to reach final concentrations of 0.8 μM. After that, the contribution of complex I on the electron flow was determined by the addition of 0.5 μM rotenone. Finally, respiratory rates were inhibited by the injection of 2.5 μg/mL antimycin A and the residual oxygen consumption (ROX) represents the oxygen consumed by the cells, not due to respiration. The maximum respiratory rates (ETS) were calculated by subtracting the antimycin resistant oxygen consumption from FCCP-stimulated oxygen consumption rates. The “leak” respiratory states, that represents the oxygen consumption in the presence of high substrate concentration but in the absence of ADP, was calculated by subtracting the antimycin resistant oxygen consumption from the oxygen consumption after Pyr+Pro addition. An injection of the oxygen enriched gaseous mixture (70% O_2_ and 30% N_2_ mol/mol) was performed once the oxygen concentration fell down below 150 nmol/mL into the O2k-chamber. This is important to avoid the potential effects of oxygen diffusion and electron transfer due to oxygen shortage during measurements [41].

Cytochrome c oxidase activity was measured polarographically at the end of the routine of respiratory analysis using 2 mM ascorbate and 0.5 mM N,N,N',N'-Tetramethyl-p-phenylenediaminedihydrochloride (TMPD), as an electron-donor regenerating system. To discriminate the oxygen consumption due to complex IV from the self-oxidation of TMPD, 5 mM of KCN was added at the end of each experiment, and cytochrome c oxidase activity was considered as the oxygen consumption rate cyanide sensitive. Since cytochrome c oxidase activity is limited by low oxygen concentrations [42], the oxygen enriched gas mixture was also injected before measuring cytochrome c oxidase activity. All oxygen consumption rates were normalized for the protein content, using another pool of heads collected in the respective hours after emergence, and quantified using the Lowry assay [24]. P<0.05; two-way mixed-effect ANOVA model was used to analyze the factors of time and sex. Tukey´s were used to correct the multiple comparisons. Tests were performed using GraphPad Prism software v.6.0.

### Citrate synthase activity

*Aedes* Rio mosquitoes (reared from F8-F10 eggs) were kept with 10% sucrose *ad libitum*, but not blood fed. The post-emergence time points tested were 2, 12, 24, 48, and 96 hours. Males and females were immobilized by chilling on ice onto a pre-cooled Petri dish and then dissected to obtain the heads (Pools of 15, N = 4). Citrate synthase activity was determined in whole cell lysates following methods described in the literature [40] with minor modifications. The heads were homogenized with a tissue grinder in a Teflon pestle in 0.05 mL of hypotonic buffer (25 mM potassium phosphate and 5 mM MgCl_2_, pH 7.2) and subsequently subjected to three freeze-thawing cycles. The enzyme activities were measured spectrophotometrically [43] at room temperature, in 75 mM Tris-HCl pH 8.0, 0.03 mM acetyl-CoA, and 0.25 mM DTNB, using a SpectraMax M3 Multi-Mode Microplate Reader spectrophotometer (Molecular Devices, California, USA). Enzyme activities were determined using samples corresponding to about 10 μg of protein. The rate of reduced coenzyme A (CoASH) production was determined using the thiol reagent 5,5’ -dithiobis (2-nitrobenzoic acid) (DTNB), which has an absorption maximum at 412 nm. P<0.05; two-way ANOVA test was used to analyze the factors time and sex. Tukey´s were used to correct the multiple comparisons. Tests were performed using GraphPad Prism software.

## Results

### Hematophagic habit manifests along the first post-emergence days

The *Aedes* Rio emerged mosquitoes exhibited a delayed response to blood feeding, becoming able to acquire blood only after 1-day post-emergence **(S1A Fig)**. Only a small number of females (13%) fed on blood on the first 24h PE, and about 36% and 61% fed respectively 48h and 96h PE.

The host seeking behavior showed that females start to seek the host with a minimum of 24h PE **(S1B Fig)**. A significant increase is observed only 96h PE when seeking behavior to a human host reaches approximately 75 cumulative visits, within a 5-minute window. Most of the females were enabled to search, find, and acquire their first blood meal upon 96h PE. Despite this, some females can feed before 96h with the same efficiency, as the ones that fed 24, 48, or 96h PE ingest equal blood amounts **(S2 Fig).**

### Differential gene expression along the post-emergence phase is time and sex-dependent

RNA-Seq transcriptome analysis was performed to understand the molecular maturation processes that occur during HGC. We evaluated the transcriptome of 72 libraries from heads and bodies of males and females, after 2, 12, 24, 48, and 96 hours PE. The time course was established based on the host seeking behavior and first blood feeding **(S1 Fig)** in agreement with the literature (reviewed by [44]). While at 2h PE females do not feed on blood, at 96h PE almost all insects are able to full blood engorgement. Using this timeframe, we were able to assess all molecular parameters during the transition period from sugar to blood meals.

A total of 1,160,614,703 raw reads were generated from heads and 1,164,028,130 from the bodies of males and females combining all time points. After removing adaptors, poor quality, and/or small reads, a total of 1,141,101,980 clean reads were obtained for the heads and 1,139,254,496 for the bodies. Almost 90% of the *Ae*. *aegypti* coding sequences (version 5.1) had aligned reads for both head and body data **(S1 Table)**.

**Fig 1:**
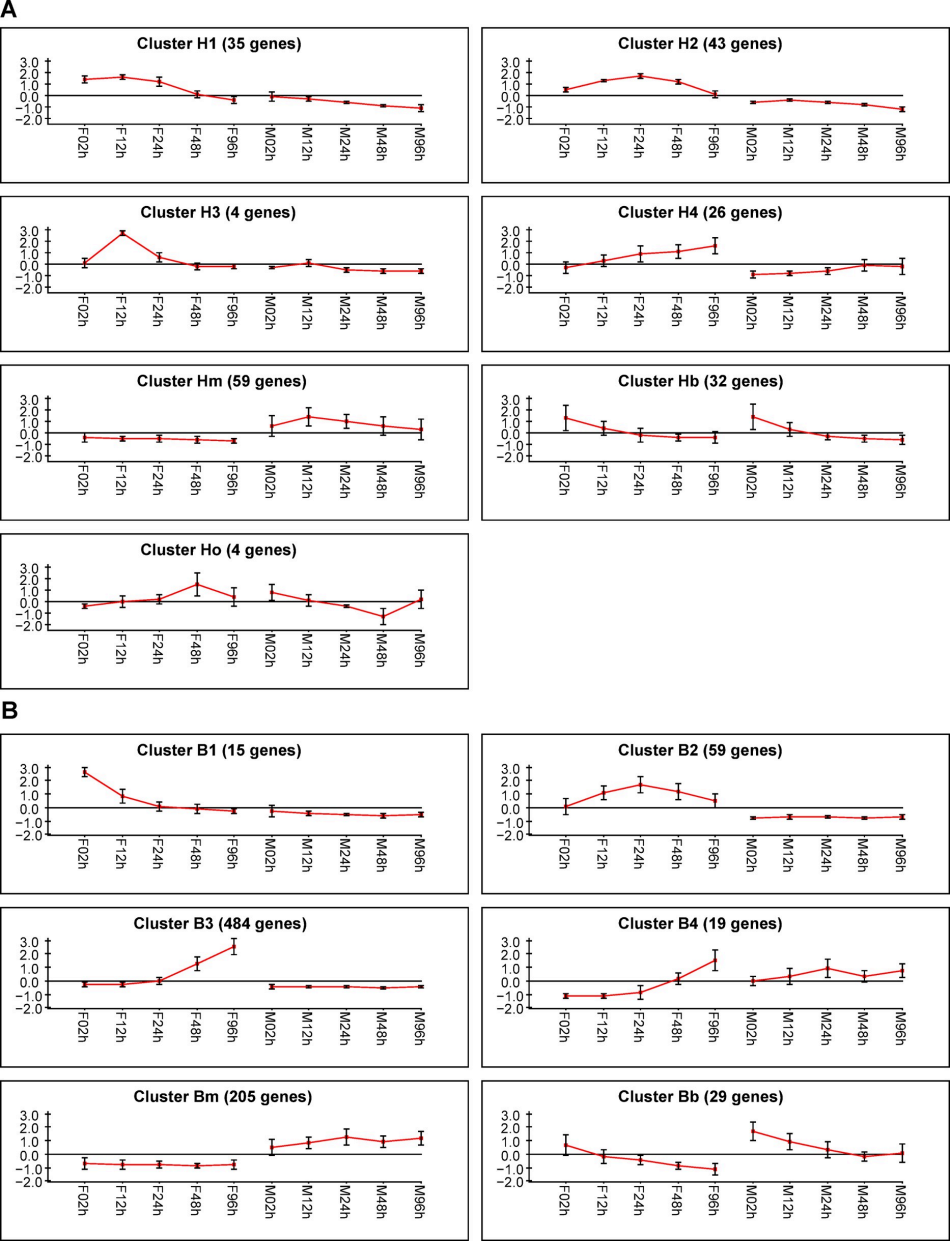
Clustering of differentially expressed genes (DEGs). Expression profiles of DEGs during the 96-h post-emergence (PE) time course. The body data generated 6 clusters (**A**) while the head data was grouped in 7 clusters (**B**). The y-axis shows normalized log2 expression values and the x-axis shows female (F) and male (M) time courses from 2 to 96 hours PE.

Differentially expressed genes (DEGs) were searched comparing the male and female time courses, considering genes modulated by time and sex (p-value <0.05). We also compared male x female data, for each of the five PE time points, using a minimum fold change of 2 and 4 (head and body, respectively) and the false discovery rate (p-value <0.05). The intersection of these statistical analyses resulted in a total of 203 and 811 DEGs identified in the head and body, respectively, in a time- and sex-dependent manner **(S2 Table)**. The expression profile clustering analysis identified female-specific time-dependent clusters showing decrease, increase, and transient modifications **(Fig 1)**. The clusters H1, H2, H3, and H4 for the head and clusters B1, B2, B3, and B4 for the body showed very little (or absent) male-specific time-dependent expression variation. Otherwise, the same clusters showed time-dependent expression variation for the females, suggesting they contain the genes that are most likely related to the HGC, since its expression is sex-dependently modulated. We also observed clusters with very low male-specific variations (Hm and Bm), with the same profile for both males and females (Hb and Bb), and with opposite profiles (Ho) **(Fig 1)**.

The female gene expression during the PE period was very dynamic, in contrast to what was observed for males. The clusters B2 (59 genes) peaked at 24h and clusters B3 (484 genes) and B4 (19 genes) showed gradual expression increase from 24h until 96h. The body clusters also presented a gradually decreased profile from 2h to 24h (B1–15 genes) **(Fig 1A)**. Some head clusters had a similar profile as the H2 (43 genes) that peaked at 24h and the H4 (26 genes) that showed gradual expression increase until 96h. Other clusters comprised the clusters H1 (35 genes) and H3 (4 genes) that had the expression peak about 12h but with different time variations **(Fig 1B).** The expression profiles were validated by qPCR **(S3 Fig)** suggesting a fine regulation of gene expression in females, compared to males.

Among the female-specific DEGs we observed an up-regulation of genes involved in the gonotrophic cycle, comprising hematophagic features such as blood ingestion and digestion, the juvenile hormone pathway, and the molecular machinery for the biosynthesis of yolk proteins.

### Expression of salivary gland anti-hemostatic genes is enhanced in females early upon emergence

**Fig 2:**
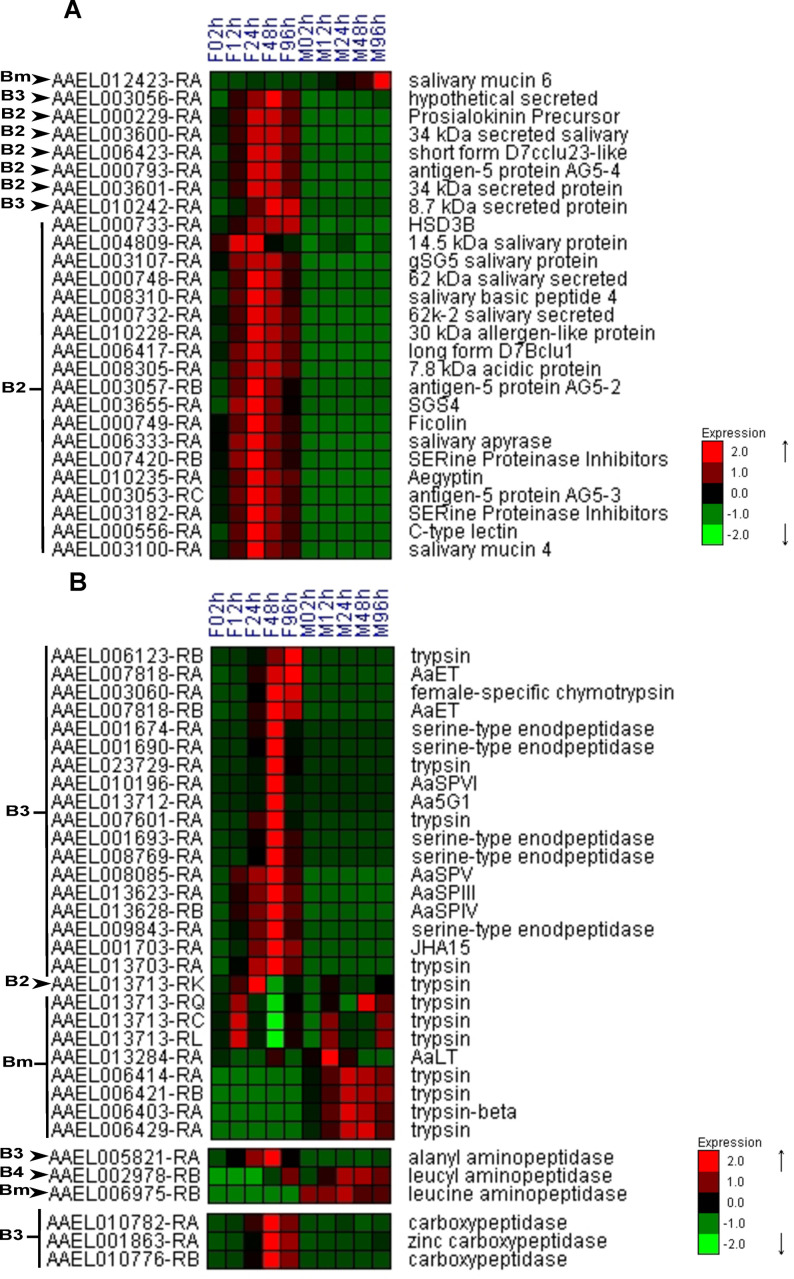
Female-specific up-regulation of salivary gland and proteases genes. Hierarchical clusterization of salivary gland **(A)** and serine proteases, aminopeptidases, and carboxypeptidases **(B)** DEGs for the body. The heatmap y-axis shows gene codes and x-axis shows female (F) and male (M) time courses from 2 to 96 hours. B2, B3, B4, and Bm are cluster names described in **Fig 1**.

The salivary glands (SG) produce molecules with anti-hemostatic properties including vasodilators, anticoagulants, platelet aggregation inhibitors, among others [20,45–49]. Notwithstanding, the feeding act is also an immune challenge, thus the saliva also contains lysozyme, antimicrobial peptides, angiopoietins, and lectins [49,50]. Most DEGs up-regulated in the female body and related to the SG **(Fig 2A)** belong to B2 and B3 clusters and were increased at 24h PE. Those include the salivary apyrase (AAEL006333), serpins (AAEL003182 and AAEL007420), c-type lectin (AAEL000556), angiopoietin (AAEL00749), antigen 5 related proteins (AAEL00793, AAEL003053 and AAEL003057), and mosquito specific families such as D7 (AAEL006417 and AAEL006423), Sialokinins (AAEL00229), aegyptins (AAEL010228 and AAEL010235) and 15–17 kDa proteins (AAEL004809).

### Expression of protease genes in females is enhanced late upon emergence

Vertebrate blood is a rich source of proteins that are digested into amino acids and subsequently used to complete the gonadotrophic cycle in adult females. The main enzymes involved in the digestion of blood proteins are endoproteolytic serine proteases, carboxypeptidases, and aminopeptidases [51,52]. Among the DEGs present in clusters B2, B3, and B4, three carboxypeptidases, two aminopeptidases, and nineteen serine proteases that were significantly up-regulated in females upon 48-96h PE **(Fig 2B)**. We also found one aminopeptidase and eight serine proteases which expression increased in males (cluster Bm) **(Fig 2B)**. From the twelve serine proteases known to be expressed in the midgut [53–58], eight were up-regulated in females at 48-96h PE in cluster B3 **(Fig 2B)**, even prior to a blood meal. They were the early phase trypsin (AaET) (AAEL007818), Aa5G1 (AAEL013712), female-specific chymotrypsin (AAEL003060), JHA15 (AAEL001703), AaSPIII (AAEL013623), AaSPIV (AAEL0013628), AaSPV (AAEL008085) and AaSPVI (AAEL010196). Although AaSPI (AAEL007432) and AaSPVII (AAEL010202) were not differentially expressed, they showed a profile similar to the B3 cluster **(S4 Fig)**. Interestingly, the late phase trypsin (AaLT—AAEL013284) did not follow the same pattern, being up-regulated in males but not in females **(Fig 2B)**. Since the AaSPII gene (AAEL008093) was removed from the last version of the *Ae*. *aegypti* transcripts gene prediction tool (5.1), its expression was not assessed.

To determine a clear pattern of serine proteases expression upon emergence, we assessed the expression profile of 90 serine proteases gene sequences previously described in the literature [59]. We observed that the expression of ~ 18% of the serine proteases analyzed was differentially up-regulated in females, mostly belonging to cluster B3. Additionally, 36% of these sequences followed the same profile of expression, increasing in females only at 48-96h **(S4 Fig)**. In combination, they represent ~ 54% of the 90 serine proteases sequences investigated, some of them with confirmed roles in blood digestion. The huge increase in protease expression observed suggests that females activate a molecular program to enable massive blood digestion. However, some of the up-regulated genes involved in proteolysis may also participate in other processes including hemostatic regulation in saliva and/or in signaling cascades.

### Previtellogenesis: Juvenile hormone signaling and ribosome biogenesis pathways

JH is an endocrine regulator that induces the heterodimerization of the JH intracellular receptor Methoprene-tolerant (Met) and its coactivator Taiman (Tai) to bind to juvenile hormone regulating elements (JHREs), modulating the expression of several genes [9] **(S5A Fig)**. The JH receptor (AAEL025915) is differentially expressed in the PE phase (B2) and Tai (AAEL023902) showed an increased profile in females, at 48-96h, for most isoforms **(S5B Fig)**.

JH signaling transcriptional repression indirectly requires the involvement of other transcription factors [16]. Hairy recruits the corepressor Groucho 1 (Gro1) down-regulating target genes [60] **(S5A Fig).** The Gro1 expression was not included in **S5B Fig** due to the very low expression level observed. It is know that Kr-h1 and Hairy work synergistically [61], we observed that Hairy expression had a more pronounced increase in females at 96h, however, Kr-h1 showed different profiles for the two isoforms, one of them increased for both sexes at 96h and the other only to males **(S5B Fig)**.

**Fig 3:**
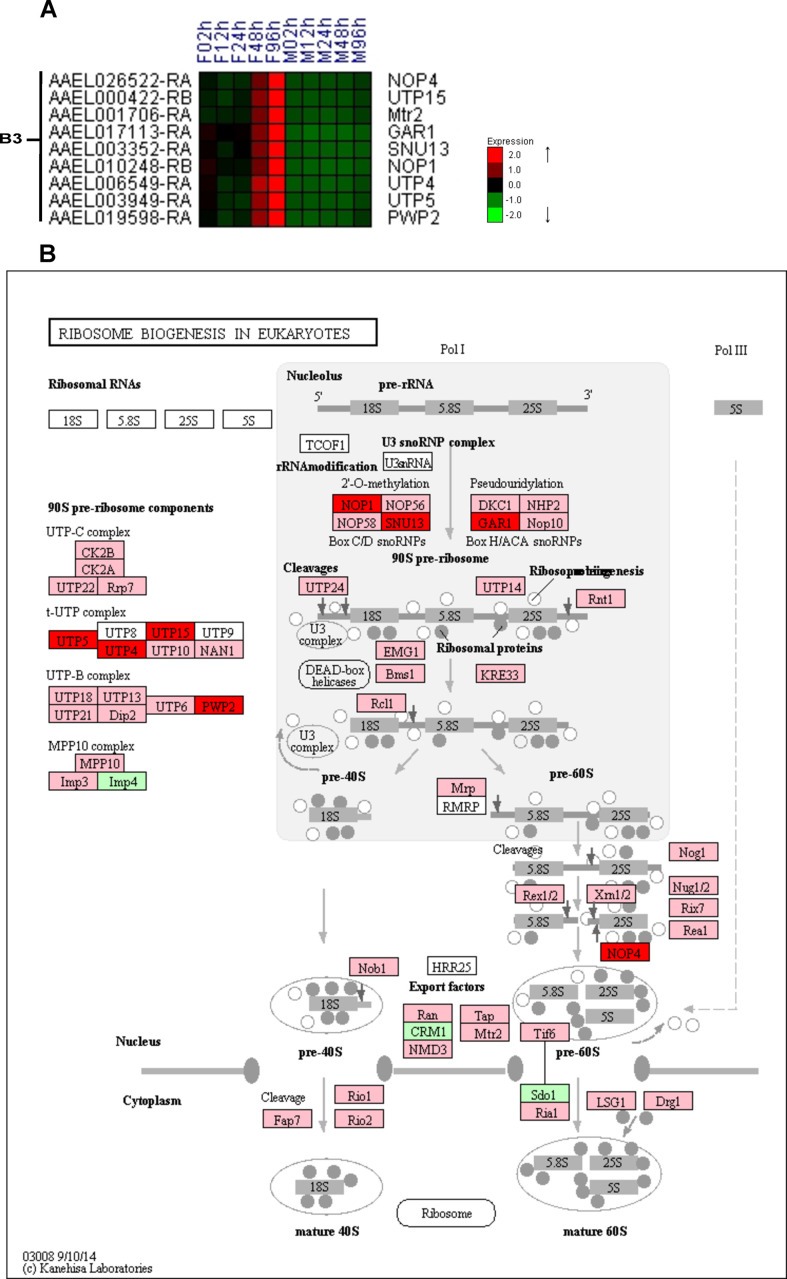
Female-specific up-regulation of the ribosome biogenesis pathway. Heatmap of differentially expressed genes (DEGs) for the body **(A)**. The y-axis shows gene codes and the x-axis the female (F) and male (M) time courses from 2 to 96 hours; B3 is a cluster name described in Fig 1. Ribosome biogenesis pathway **(B)**. DEG gene names are in red boxes (significantly upregulated in female bodies, B3 cluster), genes not DEG but with similar expression profile in pink, dissimilar or invariable profile in green and absent genes in white (Source: KEGG Mapper tool). The complete heatmap was included in **S7 Fig**.

JH plays an essential role during the HGC stimulating the FB competence for protein synthesis. The FB priming involves nucleoli enlargement, development of Golgi complex, and an increase in the number of ribosomes [11,62]. One of the genes directly modulated by the JH signaling pathway is the Regulator of Ribosome synthesis 1 (RRS1), that regulates the proteins levels of Ribosomal protein large subunit 32 (RpL32), mediating ribosomal biogenesis [12]. The expression profile of RRS1 (AAEL012185) and RpL32 (AAEL003396) was increased in females at 48-96h PE **(S5B Fig)**. The expression of the major and minor ribosome subunits genes **(S6 Fig)** and those from the ribosomal biogenesis pathway **(Figs 3 and S7)** were increased at 48-96h PE (B3). Nine genes (13%) were significantly up-regulated in female bodies (B3 cluster) **(Fig 3A)** from a pathway containing 66 genes **(Fig 3B),** and another 54 (81%) had the same expression pattern **(S7 Fig)**.

JH coordinates a series of events in the ovaries during the PE phase, including the development of a robust receptor-mediated endocytic pathway, required by the ovaries for Vg uptake [63]. Three genes regulated by this pathway were analyzed: the vitellogenin receptor (VgR), the lipophorin receptor (LpR) and a Heavy-Chain Clathrin (CHC) [64] **(S5A Fig)**. The VgR (AAEL007657) was differentially expressed (B3). The LpR (AAEL019755) has 18 isoforms in the current version (5.1) of *Ae*. *aegypti* genome gene prediction, where five isoforms were enhanced in females at 48-96h PE and eight in males (two were DEGs). We could not observe expression at a detectable level for the other isoforms. The CHC (AAEL022819) had two isoforms and both were expressed similarly in males and females **(S5B Fig)**.

### Functional enrichment analysis of the DEGs

Functional enrichment analysis was based on DEGs gene ontology (GO) classification (p-value <0.05, hypergeometric test; FDR 10% corrected Fisher’s exact test). The enriched GO terms in the body were associated with cell growth, involving cell cycle and division, DNA metabolism, and structure **(S8A Fig)**. The GO terms enriched in the head covered two major functions: (1) movement and muscle contraction; and (2) mitochondrial function, including metabolic pathways, and transport systems related to this organelle **(S8B Fig)**. The functional enrichment analysis did not find gene classes obviously related to hematophagy, however they might reflect new basic and necessary functions that support this habit. The DNA replication pathway/system and the oxidative phosphorylation pathway were analyzed in more detail further in this work.

### Increase in DNA metabolism in the female body at 48-96h post-emergence

**Fig 4:**
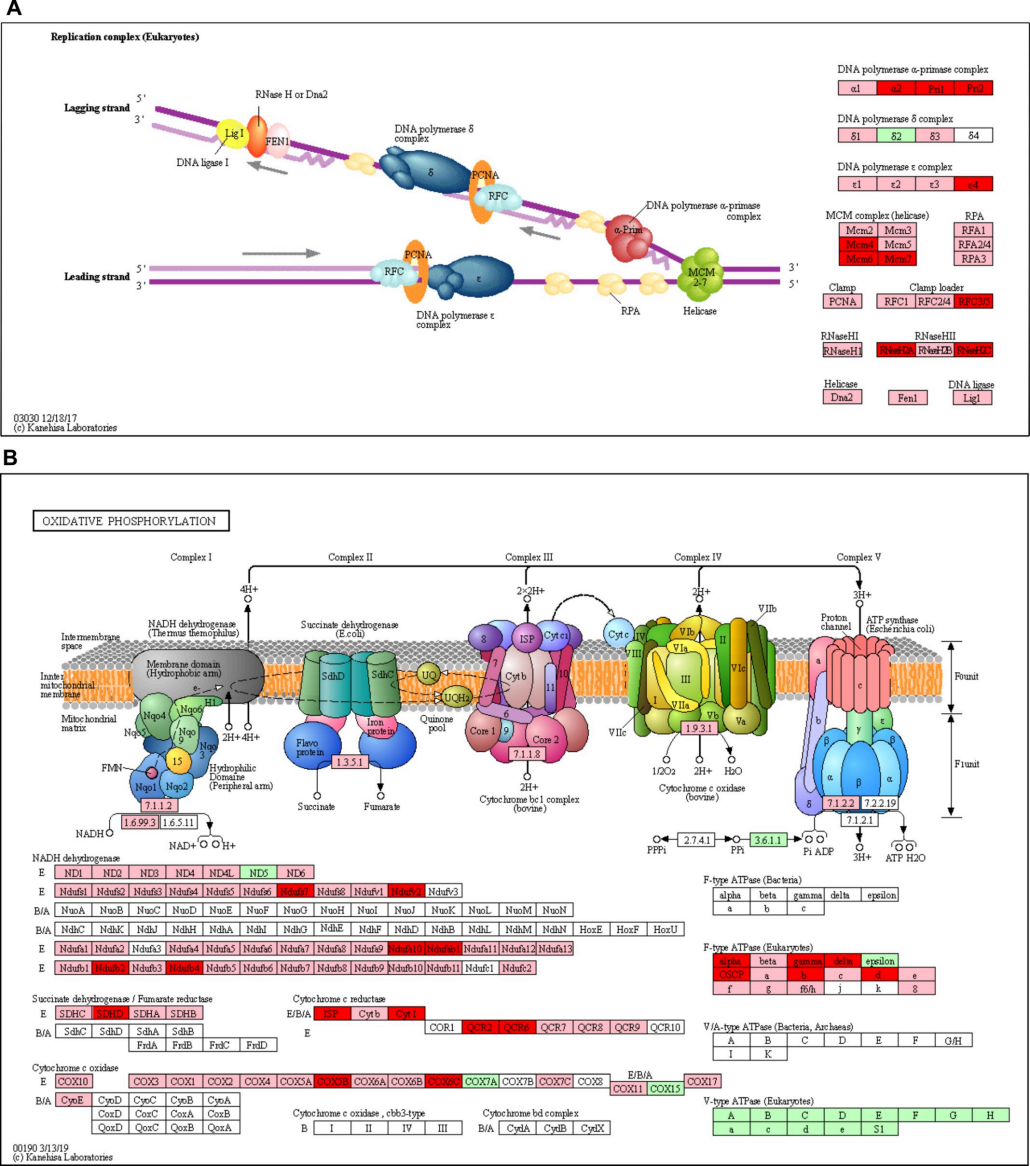
Female-specific up-regulation of DNA replication and oxidative phosphorylation genes. The DNA replication pathway was up-regulated in the female body **(A)** and the oxidative phosphorylation (OXPHOS) pathway in the head **(B)**. Differentially expressed genes (DEG) names are in red boxes (upregulated in female body, clusters B2, B3, and H2), genes not DEG but with similar expression profile in pink, dissimilar or invariable in green and absent genes in white (Source: KEGG Mapper tool). The complete heatmaps were included in **S9 and S12 Figs**.

The functional enrichment analysis showed the overrepresentation of the genetic information processing genes in the body. The classes included "nucleus", "chromosome", "nuclear chromosome", "nuclear envelope", "protein-DNA complex", "cell cycle", "DNA metabolic process", "DNA replication", "cell proliferation", "chromatin assembly" and "single-stranded DNA binding". The DNA replication pathway belongs to the DNA metabolic process and their genes were almost all more expressed in the female body over time when compared to males. This group contains approximately 31% of DEGs from Clusters B2 and B3 and more 66% following the DEG expression profile **(Figs 4A and S9A),** summing almost all genes.

We also observed an increase in gene expression of cell cycle regulators in the female body at 48-96h PE (cluster B3), such as cyclins and cyclin-dependent kinases (Cdk), and chromosome associated proteins such as histones **(S9B Fig)**. The Cyclin-dependent kinase 2 (Cdk2)—Cyclin E complex is required for G1/S transition and endoreplication in *Drosophila* [65,66]. The transcription factor E2F1 stimulates the entry in the S phase by regulating the expression of cyclin E and genes related to DNA synthesis [67]. We observed the significant up-regulation of Cyclin E (AAEL009057), and the expression increase of Cdk2 (AAEL023423) and four isoforms of E2F (AAEL022462B-E) **(S9 Fig)**.

The DNA mismatch repair pathway is responsible for the repair of mismatched base pairs during DNA replication [68] and was already described in *Ae*. *aegypti* [69]. Among its 21 genes, 5 were significantly up-regulated in females (cluster B3) and 15 more were enhanced with a similar profile **(S10 Fig)**.

Altogether, our data suggests that the cells of the female's body may be either proliferating or increasing their DNA content through the modulation of cell cycle regulators expression and the increase in DNA replication and repair pathways.

### Muscle development in females head at 2-12h post-emergence

The H1 cluster **(Figs 1B and S11)** had 18 genes (51%) associated with movement and structural functions, including cytoskeleton, muscle contraction and development. The gene ontology analysis of the head DEGs **(S8B Fig)** revealed the enrichment of "cytoskeleton" and "actin cytoskeleton" as cellular components and "muscle contraction" as a biological process. Among the proteins identified within these groups were myosins, tropomyosins, paramyosins, muscle lim proteins and myofilins. The genes coding these proteins were significantly up-regulated on females from 2 to 24h PE **(S11 Fig)**.

### Activation of mitochondrial biogenesis increases respiratory capacity and ATP-linked respiration early upon emergence in females

Among the 43 genes in cluster H2, 21 (48%) belong to the oxidative phosphorylation (OXPHOS) pathway. These genes correspond to subunits of complexes I, II, III, IV and F_1_F_o_ ATP synthase **(Figs 4B and S11)**. Remarkably, these genes were those that were significantly up-regulated in the females head over time when compared to males. We also observed that 63 genes (70%) had the same H2 group expression profile, but not differentially expressed. Only a small fraction of genes (6%) in the OXPHOS pathway presented an expression profile that did not fit the H2 cluster **(Figs 4B and S12)**. Globally, we observed a female-specific expression increase of genes involved in OXPHOS pathway along maturation.

**Fig 5:**
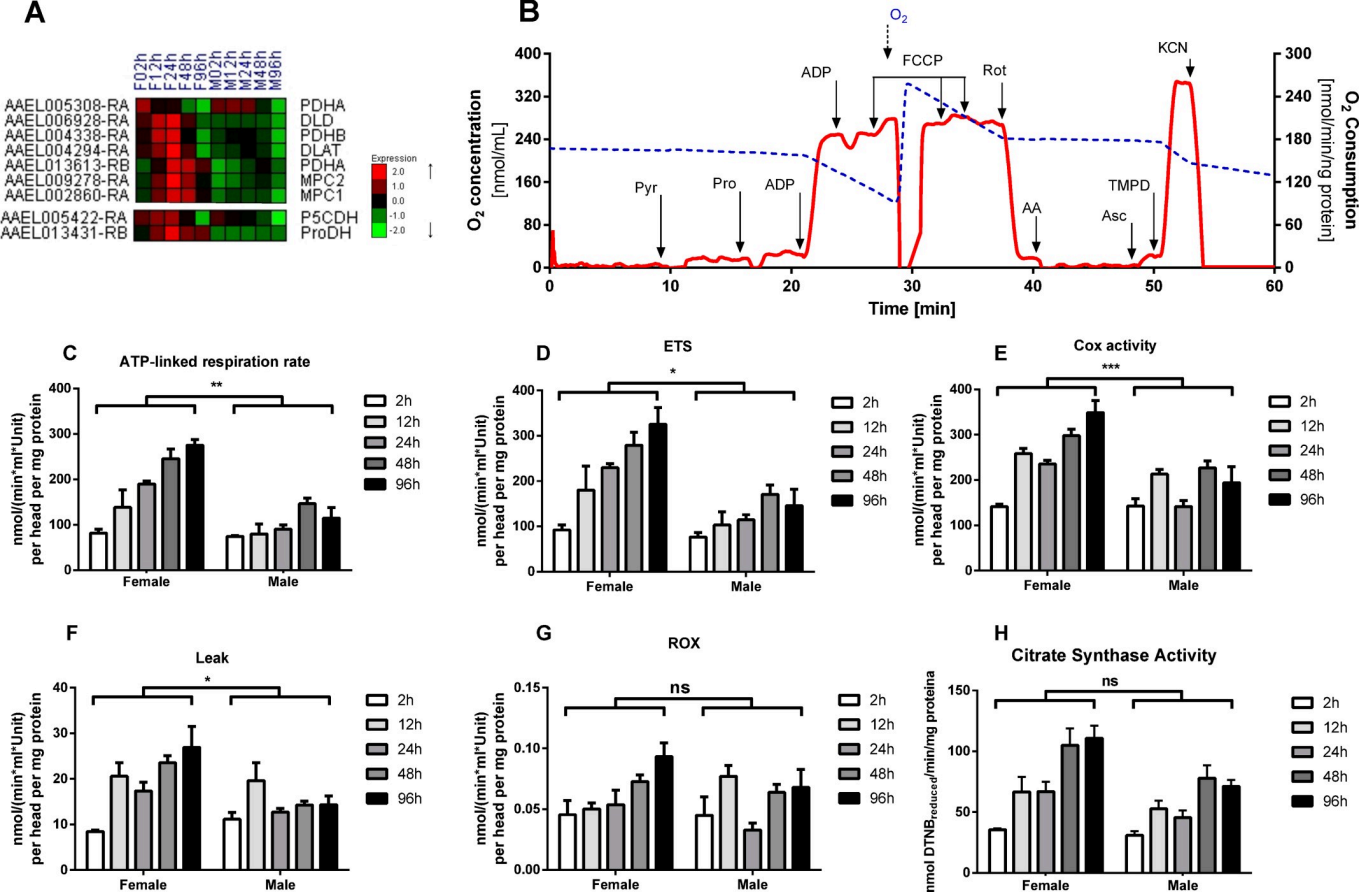
Increased expression of genes involved in mitochondrial processes drive respiratory rates and OXPHOS. Heatmap of genes related to pyruvate and proline oxidation **(A)**, the y-axis shows gene codes and x-axis shows female (F) and male (M) time courses from 2 to 96 hours, details in **S13A Fig**; typical traces of oxygen consumption rates (OCR) **(B)**, the OCR (red line) and concentration (blue line) were obtained from mechanically permeabilized *Aedes* Rio female heads at 24 hours PE using Pyr+Pro as substrates; oxygen consumption linked to ATP synthesis (OXPHOS) **(C)**; maximum respiratory rates (ETS) **(D)**; Cytochrome c oxidase (COX) activity **(E)**; Leak represents the oxygen consumption in the presence of high substrate concentration but in the absence of ADP **(F)**; residual oxygen consumption (ROX) **(G)**; citrate synthase activity **(H)**. Bar graphs show mean (SEM) for male and female at different PE time points. Interaction p-value between sex and time factors (two-way ANOVA) are displayed above the bars. ns = not significant; *p-value <0.05; **p-value <0.01; ***p-value <0.001.

Since the expression of genes involved in OXPHOS pathway was remarkably increased in female heads along PE, we next wondered whether this would be reflected in a gain of metabolic function. We then determined oxygen consumption rates (OCR) using pyruvate and proline (Pyr+Pro) as substrates along the same time course in mechanically permeabilized male and female heads **(Fig 5)**. The reason to use these substrates lies in the fact that respiration coupled to ATP synthesis in *Ae*. *aegypti* flight muscle mostly uses proline and pyruvate to sustain respiration rates [38,40]. Indeed, we observed a trend towards an increase in expression of genes coding for pyruvate/proline transport and/or oxidation in the female upon PE **(Figs 5A and S13A)**. Examples include proline dehydrogenase, subunits of pyruvate dehydrogenase complex, and mitochondrial pyruvate carrier **(Fig 5A)**. The expression profile of these genes was very close to the H2 cluster, the same found for almost all genes of the OXPHOS pathway.

**Fig 5B** shows a representative trace of oxygen consumption of mechanically permeabilized *Ae*. *aegypti* female heads using Pyr+Pro as substrates. The oxygen consumption coupled to ATP synthesis (ATP-linked) significantly increased in female heads over time **(Fig 5C)**. This strongly indicates that increased expression of OXPHOS components in females head upon PE reflects in improved energy supply by mitochondria. The maximum respiratory rates (ETS), induced by uncoupling of respiration from ATP synthesis, also increased significantly in females over PE time points **(Fig 5D)**. Interestingly, the slight differences between ATP-linked and ETS respiratory rates indicate that ATP synthesis through OXPHOS requires almost the maximal capacity provided by mitochondria, similarly to flight muscle mitochondria [38,40]. Cytochrome c oxidase (COX) activity **(Fig 5E)**, leak **(Fig 5F)**, and residual respiratory rates **(Fig 5G)** followed the same trend, significantly increasing in females' heads over time. Remarkably, respiratory rates in males´ heads were not significantly altered upon PE for any of the mitochondrial metabolic states investigated **(Fig 5)**. Therefore, increased expression of OXPHOS pathway genes **(Fig 4B)** resulted in a female-specific gain of function in *Ae*. *aegypti* head in the first days after emergence, underscoring the key role of OXPHOS to HGC **(Fig 5)**.

The simultaneous increase in all mitochondrial metabolic states assessed is suggestive of induction of mitochondrial biogenesis. We then measured citrate synthase (CS) activity, as a proxy of mitochondrial content [70]. A two-step increase in CS activity was observed in female heads: the first one at 12-24h PE and a second and higher one at 48-96h PE. Curiously, the pattern of increased CS activity is sex-independent, although at the end of the maturation process (96h) was significantly higher in females compared to males **(Fig 5H)**. Despite this, the overall comparison between the female and male CS time curves was not significant. We then considered whether mitochondrial biogenesis would be responsible for the differences observed in respiratory rates, by normalizing OCR by their respective CS activities in each sex group and time points. **S13B–S13F Fig** show that all significant time-sex-related differences observed in **Fig 5** were completely abolished, regardless the mitochondrial metabolic state. Together, these results indicate that the maturation process upon emergence in females´ head involves the activation of mitochondrial biogenesis that ultimately increase mitochondrial content, respiratory rates and energy provision by OXPHOS.

The expression of genes involved in mitochondrial electron transport system, heme biosynthesis and mitochondrial DNA metabolism are regulated by transcriptional factors such as peroxisome proliferator-activated receptor-gamma coactivator 1 alpha (PGC-1ɑ), mitochondrial transcription factor A (TFAM) and dimethyladenosine transferase 2 (TFB2M) [71–73]. Interestingly, the *Ae*. *aegypti* orthologues of PGC-1ɑ (AAEL003768), TFAM (AAEL013643) and TFB2M (AAEL012582) were up-regulated in female heads earlier than those involved in OXPHOS pathway **(S14 and S12 Figs)**. We also observed the expression of the first enzyme of the heme biosynthetic pathway, 5-aminolevulinate synthase (ALAS2—AAEL007787), that had two of three isoforms with a similar expression profile as the OXPHOS pathway genes, a transient increase peaking at 12-24h **(S14 Fig)**. Therefore, we propose the hypothesis that activation of specific transcription factors involved in mitochondrial biogenesis is an early event that results in later increases in expression of OXPHOS pathway components, and ultimately enhancing respiratory rates and energy supply through the oxidative phosphorylation.

## Discussion

The understanding of the hematophagic and gonotrophic capacitation (HGC) as a maturation process that occurs in newly emerged females is fundamentally important to conceptually identify specific new targets for vector control. Previous transcriptomic, microarray and proteomic studies have studied the post-emergence (PE) phase in females, revealing altered metabolic pathways in midgut, fat body and salivary glands [16–20,74], but just the mosquito antenae transcriptome compared gene expression based both on sex and time [21]. Notwithstanding, the available knowledge did not consider the sexual dimorphism in the time context of PE phase and were mostly focused on the fat body. To fill this gap we performed time-dependent molecular and biochemical analyses to comprehensively identify mechanisms involved in *Ae*. *aegypti* HGC comparing both sexes upon emergence. Our transcriptomic analyses showed that genes involved in blood feeding were significantly up-regulated exclusively in females even prior to the first blood meal, in a time-dependent manner. We observed that cell growth and DNA metabolism related genes were more expressed in the female body than in males. We also showed that the OXPHOS pathway and consequently the respiration rates enhanced in females head, compared to the male, due to a remarkable increase in mitochondrial content. Collectively, the data presented here support the notion the activation of a sex-biased program in female´s head involving mitochondrial biogenesis and OXPHOS activity is a key mechanism underlying HGC process. Although the mechanisms involved in the activation of mitochondrial biogenesis genes remain elusive, we envisage that targeting such mechanisms would represent new avenues for vector control strategies.

The *Aedes* Rio colony started recently from field mosquitoes captured in Rio de Janeiro state, Brazil. The assessment of the females blood feeding and host seeking behaviours showed that both started at 24h and increased until 96h **(S1 Fig)**. These results are in agreement with reported data. Early studies determined the pattern of the first blood-feeding for *Ae*. *aegypti*, where most of the individuals (~50%) acquired the first blood meal upon 24–48 h PE (reviewed by [44]. The host seeking behavior has been described after 18-24h PE [75].

Here we observed cohorts of gene expression unique to females in the PE phase in the head and body **(Fig 1)**. It is remarkable that each cluster was mainly associated with a specific function/system, except cluster B3 that contained 484 genes, and therefore more functions. The H1 cluster **(Fig 1B)** was associated with movement function, with proteins related to the cytoskeleton and to muscle contraction. The head actually has several muscles associated with sensory organs and mouthparts, including the salivary pump that connects the esophagus, neck and thorax [44]. From 2 to 24h PE there was an up-regulation of proteins associated with muscular function **(S11 Fig)** previously to the blood-feeding behaviour start **(S1 Fig)**, suggesting that the head muscles development after the adult emergence is necessary for the blood ingestion. The decision of using the whole head and decapitated body prevented the identification of the origin tissues of DEG in some cases, but also avoided *a priori* exclusions that allowed findings like this related to the muscular tissue. The H2 cluster **(Fig 1B)** had a very marked relationship with the OXPHOS pathway **(Fig 4B)**, almost 50% of the H2 genes coded proteins to this pathway. The salivary protein coding genes are in the cluster B2 (16 genes, representing 27% of the cluster) and cluster B3 (7 genes, representing 1.5% of the cluster) **(Figs 1A and 2)**. The cluster B3 **(Fig 1A)** was not only the biggest cluster with 484 genes, but also the most functionally diverse. This particular cluster contained proteases, cell cycle, DNA replication and repair, ribosome and ribosome biogenesis, transcription factors and signaling genes, among others **(Figs 3–5 and S4–S8)**.

The remarkable changes in gene expression that females experienced upon emergence can be observed by the inflection present at the 24h time point in almost all cluster gene expression curves (**Fig 1**: B1, started baseline at 24h; B2 and H2, sine wave top; B3 and B4, started expression increase). These gene expression changes at 24h were synchronized and very closely related to the female ability to start to feed on blood at this time (reviewed by [44]) **(S1 Fig)**. Among the genes found up-regulated in females were those related to blood feeding, so we decided to evaluate the expression of genes previously associated with the gonotrophic cycle, such as salivary proteins, proteases, ribosome biogenesis and JH signaling pathways **(Figs 2–4 and S4–S6)**.

### Salivary genes were up-regulated in females as preparation for blood meal intake

Salivary sex-specific proteins of 2 to 5 days old adult *Ae*. *aegypti* were already described [20] and our data confirmed the up-regulation in the female body of 25 salivary genes from the 84 described (B2 and B3 clusters, **Fig 2A**). The difference probably was due to the samples, while we used the mosquito whole body (that contains the salivary glands), Ribeiro used only the salivary glands. One example of up-regulated gene is aegyptin (AAEL10235-RA), the coded protein binds to collagen, inhibiting platelet aggregation and adhesion. Its down-regulation promotes longer probing time and reduction on feeding success [48]. All of the salivary up-regulated genes have the same transient expression, peaking at 24h or 48 PE. Their maximum production matches the time that the females are enabled to acquire a blood meal, showing again that the saliva is obviously a piece of the HGC.

### Proteases involved in blood digestion are highly up-regulated in females body

After the ingestion of a blood meal, proteases, especially trypsins, play an important role in protein digestion [52,76]. The literature has many descriptions of trypsins that have their protein level/activity triggered by a blood meal, such as AaET, AaSPI, AaSPVI, AaSPVII, AaLT, and female-specific chymotrypsin [52–55,57,77]. Although we did not include blood-fed females, we could find 18% of all proteases (listed by [59]) significantly up-regulated in the body at 48-96h (B3 cluster) and other 36% with an expression increase **(Figs 2B and S4)**. These results suggest that some proteases are up-regulated by the HGC, previously to the first blood feeding, complementary to the common-sense knowledge of protease regulation by the blood diet ingestion.

Two serine proteases with trypsin-like activities account for the majority of the blood meal digestion [52,53]. AaET, that shows an intense proteolytic activity between 1 and 6h after a blood meal, and AaSPVI, that belong to the 5G1 family, has it's main activity (75%) from 12-36h after a blood meal [52,53]. Evidence indicates that AaET is transcriptionally regulated by JH and translationally regulated by a blood meal [78,79]. Our results suggest that this feature could not be exclusive from AaET since the proteases AeET (AAEL007818), female-specific chymotrypsin (AAEL003060), AaSPVI (AAEL010196) and AaSPVII (AAEL010202) had expression peaking at 48h PE **(Fig 2B)** and were already described to have their activity increased by the blood meal. Despite the classical role of serine proteases in midgut blood digestion, we cannot rule out their potential involvement in key biological functions in other tissues. For instance, it was already described the presence of serine proteases in the salivary glands of adult females and it was suggested they play a role in immunity [48]. The male expression increase of several trypsins analyzed **(Fig 2, cluster Bm)** was not an expected result. For example, the late phase trypsin (AaLT—AAEL013284) is known as a midgut-specific protein that increases in content and activity after a blood meal in females [79,80]. Interestingly, it was significantly up-regulated in males after the emergence, raising questions about its biological role and tissue origin on the males, that are not under HGC.

### Juvenile Hormone signaling differs between female and male mosquitoes

The JH drives the shift of unfed *Culex nigripalpus* females from nectar to blood-host odour [81]. This hormone acts regulating gene expression during the PE phase, where the genes/proteins involved in the signaling were already identified in the female fat body and ovaries [82,83]. Our results revealed these genes have expression differences between the female and male mosquitoes **(S5B Fig)**. One isoform of the key gene Met (AAEL025915-RC) is significantly up-regulated in the female body. Some isoforms of Tai (AAEL023902) and Hairy (AAEL027674) were also increased at 48-96h PE in the female body, but others showed expression on the male body. The gene Kr-h1 was previously classified as late PE phase gene [16]. We observed the same late expression pattern to both sexes for Kr-h1 isoform A (AAEL002390-RA) while the other isoform was increased only in males (AAEL002390-RB) **(S5B Fig).** These time-sex differences highlight the relevance of JH signaling pathway for the female HGC and also are closely related to the striking difference in the gene expression clustering between the sexes **(Fig 1)**.

An important molecular machinery regulated by JH signaling is the ribosome biogenesis pathway. This machinery is important for vitellogenesis, including the synthesis of Vitellogenin (Vg), a major yolk protein. The female time-dependent expression increase of ribosomal genes was shown on the fat body [12]. Here, we revealed that the expression of almost all genes of the ribosome biogenesis, including the ribosome subunits themselves have significant up-regulation or increased expression not only based on time, but also on sex **(Figs 3 and S6 and S7)**. The vitellogenin receptor (VgR) has a known expression profile in the ovary by Northern blot [84]. Our observations confirmed the VgR (AAEL007657) female expression profile, significantly up-regulated in the body **(S5B Fig)**, due to the vitellogenesis preparation.

The lipid metabolism is also related to the JH signaling [74]. In *Ae*. *aegypti* female fat body there is a known TAG accumulation until 72h PE and a decrease in **β**-oxidation and lipid catabolism [74]. Here, we observed sex-specific expression increase of the lipophorin receptor isoforms (LpR) (AAEL019755) **(S5B Fig)**. The five isoforms more expressed in females might be related to the fat body lipid stock increase and/or the oocytes preparation for the uptake of lipophorin during vitellogenesis [64]. In males, the other eight isoforms of LpR had increased expression compared to females, pointing that the lipid distribution may differ strongly between sexes in the first days PE **(S5B Fig)**.

### Cell cycle control during previtellogenesis

Cells in endoreplication suppress mitosis and cytokinesis repeating S and G phases successively, increasing their size and ploidy [85]. In *Locusta migratoria*, the endoreplication in fat body is regulated by JH signaling that induces the transcription of chromosome maintenance (Mcm) genes Mcm4 and 7, cell-division-cycle 6 (cdc6), cyclin-dependent kinase 6 (Cdk6) and E2F [86,87]. The G2 to M phase progression can be avoided down-regulating the expression of the Mitotic cyclin-dependent kinase (M-CDK), also known as cdc2/CDK1, or their protein levels by anaphase promoting complex (APC) ubiquitin-mediated proteolysis of cyclins or cyclin kinase inhibitors [65].

We observed the significant up-regulation of Mcm4 (AAEL10086), Mcm7 (AAEL000999), and the expression increase of cdc6 (AAEL010855), Cdk4/6 (AAEL001407), and APC complex proteins **(S9 Fig)**. Besides the regulation, the DNA replication pathway itself had 97% of its genes significantly up-regulated or with enhanced expression in the female body **(Figs 4A and S9A)**. Other proteins associated with the cell cycle regulation and progression were also up-regulated, such as cyclins, cyclin-dependent kinases, and structural proteins that associate with the DNA such as histones **(S9B Fig)**. These observations suggest the control of the G1 to S progression, possibly avoiding M phase, which could be a sign of endoreplication engagement [88,89]. This regulation is crucial to the HGC, and some changes can severely impact those processes, such as the depletion of Cdk6 and E2F that are known to reduce the endoreplication in the fat body arresting oocyte maturation [90].

### Increasing of the DNA mismatch repair pathway and decreasing of apoptosis

The endoreplication process is also related to changes on the DNA maintenance and apoptosis pathways in eukaryotic cells [91]. The key genes of the DNA mismatch repair pathway (MMR) activation MLH1 (AAEL005858) and PMS2 (AAEL026487) were significantly up-regulated in the female body together with the expression increase of 71% of the other genes from this pathway **(S10 Fig)**. They followed the same expression pattern of the endoreplication genes already mentioned **(S9 Fig)**, indicating a possible enhancement of the DNA replication and repair in the females in the first hours PE. The apoptosis is controlled to avoid the death of the polyploid cells [91]. At 48–96 PE, one isoform of the inhibitor of apoptosis 1 gene (Ae*iap*1) (AAEL009074-RE) was significantly up-regulated in females, while the other isoforms were relevant to males (AAEL09074-RB/AAEL009074-RC) **(S9B Fig)**. The core caspases activated during apoptosis, AeCASP7 (AAEL012143) and AeCASP8 (AAEL014348) [92] are down-regulated in females at 48-96h PE **(S9B Fig)**. The enhancement of Ae*iap*1 and decrease of AeCASP7 and AeCASP8, suggests that apoptosis pathway was down-regulated, corroborating with the hypothesis of endoreplication activation.

Altogether, our data suggests that endoreplication is activated. Considering the pathways we found regulated (ribosome biogenesis, ribosome subunits, JH signaling, MMR, DNA replication, cell cycle regulators, and apoptosis) **(Figs 3 and S5–S9)** and the literature insights in *L*. *migratoria* and *Drosophila*, we suspect the fat body and midgut as possible sights of endoreplication [88,90]. The polyploid phenotype provides an advantage to synthesize and secrete a huge amount of macromolecules [85]. We show that during HGC there is an expression increase in the machinery for protein synthesis (ribosome biogenesis and ribosomes) triggered by JH signaling pathway, possibly supporting the known fat body huge and rapid biosynthesis after the first blood meal.

### Muscle changes in females' heads

Miofilin is a core protein that interacts with myosins sub filaments that is required for the correct assembly of thick filaments in insects striated muscles [93]. Flightin is essential for the stability of thick filaments in the indirect flight muscle of *Drosophila* by regulating the filament and sarcomere length [94,95]. It also contributes to stiffness and performance of oscillatory work [96]. The contraction in thin filaments striated muscles are associated with the activation of the tropomyosin-troponin complex through Ca^2+^ binding [97].

Here we observe a significant up-regulation of myofilin (AAEL001082), flightin (AAEL004249), myosin light chain 1 (AAEL012207), myosin heavy chain (AAEL026217), troponins I, C and T (AAEL010850, AAEL06572 and AAEL026967) and tropomyosin (AAEL002761) in females at 2-24h PE **(S11 Fig)**. Notwithstanding the mentioned proteins, the H1 cluster **(Fig 1)** sums 51% of its proteins associated with the actin cytoskeleton and muscle contraction, as identified by GO enrichment **(S5B Fig)**.

The sex differences in gene expression observed in the head suggest that females muscle thick filaments had improvement assembly, stability and/or strength. There are many muscles at the mosquito head, they allow movements in sensory organs and mouthparts. Likewise the diet intake and saliva injection are coordinated by the cibarial and pharyngeal pumps that contract in alternation, oscillating at a frequency of 4.3 Hz [98]. The physiological differences between sexes demands from females more sensory and feeding challenges, since they need to find the blood source and suck a fluid that is more viscous than sap, needing higher pump power [99]. It is important to notice that the higher female expression of the genes related to muscular function **(S11 Fig)** occurs during HGC, previously to the observation of the first engagement in a blood meal and may be needed for it but further investigation is necessary to define their relation.

### Mitochondrial biogenesis triggers an increase in mitochondrial content and respiratory rates

A remarkable observation was the early increase in expression of mitochondrial biogenesis transcription factors peroxisome proliferator-activated receptor-gamma coactivator 1 alpha (PGC-1ɑ; AAEL003768), the mitochondrial transcription factor A (TFAM) (AAEL013643) and dimethyladenosine transferase 2 (TFB2M) (AAEL012582) **(S14 Fig)**. PGC-1ɑ is known to induce mitochondrial biogenesis by increasing the expression of genes involved in electron transport system, heme biosynthesis, mitochondrial DNA (mtDNA) transcription and replication [71–73]. Interestingly, the expression of PGC-1ɑ occurred early to the OXPHOS pathway components, suggesting a regulatory role in our model. Similarly, the expression of TFAM and TFB2M, which are key activators of transcription and replication of mtDNA [100], exhibited an expression profile similar to PGC-1ɑ **(S14 Fig)**. On the other hand, the expression of nuclear respiratory factors 1 and 2 (NRF1/2) did not follow this trend, since their expression was reduced in female heads **(S14 Fig)**. Indeed, we observed a significant up-regulation of electron transport system genes **(Fig 4B)**. We also observed expression increase of many other genes involved in OXPHOS including mitochondrial pyruvate carrier 1 and 2, proline dehydrogenase, TCA cycle enzymes, as well as many components of the electron transport system **(Figs 4B, 5A and S12)**. Importantly, these events culminated with a striking increase in mitochondrial content, respiratory rates and ATP-linked respiration **(Figs 5 and S13)**. Although the mechanisms that trigger PGC/Tfam expression remain elusive, the activation of a concerted program towards energy provision through OXPHOS in female heads is clear. Conceivably, increased ATP demand is synced with the start of the blood feeding and host seeking behaviors **(S1 Fig)** during the HGC. The aforementioned muscle differences together with the female sensory neuronal activity (required for host seeking) may be the source of the extra energy demand that could be met by a more robust energy provision process (OXPHOS).

The synapses require a huge energy requirement. The regulation of ion contents across the membrane, the synaptic vesicles trafic, the neurotransmitters secretion and reuptake are very high energy demands, that is provided by the mitochondrial metabolic activity [101]. Additionally, the mitochondria responds to energy requirements and are transported through the neuron as needed [102–105]. Specialized cells, such as the photoreceptors cells, have a high mitochondrial density that can be redistributed according to energy demand [106]. In newly emerged females, this energy power system might be adjusting to be able to support the perception of huge amounts of new environmental clues, and contributing to the maturation of the host seeking behavior, that occurs at 24h PE **(S1 Fig)** during HGC. More research needs to be done in order to determine the tissue specificity of the phenotype and the relationship with the blood feeding and host seeking behaviors.

### HGC and vector control

The hematophagic and gonotrophic capacitation (HGC) is a female-specific process that takes place in the first days after the emergence, resulting in a large number of differentially expressed genes (DEG) peaking at different times during this period. We envisage that identification of critical molecular mediators of HGC as potential targets to be explored in the future, representing innovative and valuable tools aiming the reduction in *Ae*. *aegypti* fitness and vector control. In this sense, we suggest a small set of genes involved in HGC considering those DEG with the strongest sex-dependent expression (highest in females and lowest in males) as the most interesting ones to be targeted by available genetic interventions. These include the salivary antigen-5 protein AG5-4 (AAEL000793-RA) and the salivary basic peptide 4 (AAEL008310-RA), both with expression levels very close to Aegyptin (AAEL010235-RA). Among the proteases, JHA15 (AAEL001703-RA) and female-specific chymotrypsin (AAEL003060-RA) are good candidates as both have expression levels quite close to AaETs (AAEL007818-RA-B). The tubulin beta chain gene (AAEL002848-RA—Muscle group), RFC3/5 (AAEL007581-RA and AEL009465-RA—DNA repair group), Cyclin B (AAEL010094-RA—Cell cycle group) had the highest and significant expression difference between sexes. Notwithstanding, genes with sex-specific expression can also highlight CRISPR-Cas9 gene disruption candidates, like the Histone H3 (AAEL019635-RA) which is barely expressed in males, and lipophorin receptor (AAEL019755) that have 13 isoforms predicted. The transcripts *RA* and *RP* were DEG on the male (cluster Bm) while the transcript *RO* despite not DEG was the one with the highest female expression. Finally, despite clear sex-dependent increases in mitochondrial metabolism gene expression toward the females were observed **(S12 Fig)**, the overall changes were not as high as those identified in other mechanisms. For this reason, we could not suggest any particular mitochondrial mechanism to be further explored as a valuable target at this point.

The present study provides insightful information about sex and time effects on the gene expression dynamics during the first four days after mosquito emergence. In the female head, at 2-24h PE (cluster H1) there was an up-regulation of genes related to muscle development and contraction, suggested to be associated with the feeding/sucking pumps strengthening for the blood ingestion, denser than sap. At 24h PE (cluster H2) there was the up-regulation of the oxidative phosphorylation pathway. Functional assessment of mitochondrial function revealed a gradual increase in respiration capacity, ATP-linked respiration and mitochondrial content until 96h, suggesting the activation of mitochondrial biogenesis in heads is a required step towards blood sucking habit. The increase in ATP offer could supply the intense muscle contraction, brain activity and chemosensory responses required before or during the blood feeding. Considering the female body, at 24h PE (cluster B2) there was an increase in salivary gland genes that allow the blood ingestion by their antihemostatic properties. Several pathways were enhanced at 48-96h PE (cluster B3). The huge increase in trypsins is probably related to blood digestion, and the cell cycle, DNA metabolism, ribosome biogenesis, and juvenile hormone signaling pathways are possibly related to polyploidization (endoreplication) and intense biosynthesis preparation for the vitellogenic stage. Our data showed a strong relationship between the up-regulation of female-specific genes along the first days PE and the hematophagic capacitation and maturation process, unraveling not only new mosquito biological aspects but also new potential targets for vector control.

## Supporting information

S1 Fig *Aedes* Rio females take at least 24 hours to mature the host seeking behavior and take the first blood meal. **(A)** Percent females freely feeding on an offered artificial blood meal; and **(B)** Cumulative number of female visits to the net in the area exposed to a human arm. The post-emergence time points tested were 2, 12, 24, 48, and 96 hours. Bars upper and lower whiskers represent the highest and lowest observations. The line inside the bar represents the median. (Kruskal-Wallis corrected one-way ANOVA comparing the hours post emergence; (a) P<0.005 (b) P<0.05). N = 5).
(TIF)

S2 Fig Blood intake by *Aedes* Rio females during post-emergence phase. Total protein amount was quantified per midgut of fed and unfed females after offering an artificial blood meal for 30 minutes. "X", at 2 and 12h PE none mosquito took the blood meal. (Tukey’s corrected one-way ANOVA; *** P<0.0001. N = 4).
(TIF)

S3 Fig Validation of female-specific expression profiles by qPCR analysis. One gene was selected to represent the profile of clusters: B1 (A), B2 (B), B3 (C), H1 (D), and H4 (E). Bar graphs show mean (SEM) for males and females. The post-emergence time points tested were 2, 24, and 96 hours. Interaction p-values between sex and time factors (two-way ANOVA) are displayed above the bars. ns = not significant; *p-value <0.05; **p-value <0.01, ***p-value <0.001.
(TIF)

S4 Fig Serine proteases expression profile. Hierarchical clusterization of the serine protease genes in the post-emergence time points of 2, 12, 24, 48, and 96 hours. Heatmap y-axis shows gene codes and x-axis the female (F) and male (M) time courses from 2 to 96 hours. B2, B3, and Bm are cluster names described in Fig 1. Up-regulated genes are highlighted by red arrows for females and blue arrows for males.
(TIF)

S5 Fig Juvenile hormone (JH) signaling pathway and its target genes in the fat body and ovary. Summarized juvenile hormone (JH) signaling pathway **(A)** The JH biosynthesis occurs in the corpora allata (CA), a pair of endocrine glands with neural connections to the brain. There is a relevant JH increase in hemolymph 12h PE leading the fat body (FB) priming and maturation of oocytes for vitellogenesis. JH has an intracellular receptor Methoprene-tolerant (Met), which binds to Taiman (Tai), this heterodimer binds to Juvenile hormone response elements (JHREs) modulating gene expression. Hairy and Kruppel homolog 1 (Kr-h1) are transcription factors that will regulate the expression of other genes. In the FB, this pathway will activate Ribosome Biogenesis Regulator 1 Homolog (RRS1) and Ribosomal protein L32 (RpL32), required for Ribosome biogenesis pathway and subsequently the production of vitellogenin (Vg). In the ovary, the activation of this pathway will regulate the expression of genes such as vitellogenin receptor (VgR), Heavy-Chain Clathrin (CHC) and the lipophorin receptor (LpR), related to the uptake of vitellogenin (Vg); Heatmap of Juvenile hormone signaling pathway and the cited target genes for the body **(B)**. Heatmap y-axis shows gene codes and x-axis shows female (F) and male (M) time courses from 2 to 96 hours. B2, B3, and Bm are cluster names described in Fig 1. Up-regulated genes are highlighted by red arrows for females.
(TIF)

S6 Fig Ribosome large and small subunits expression profile. Differentially expressed genes (DEG) names are in red boxes (up-regulated in female body—group B3), genes not DEG but with similar expression profile in pink, dissimilar or invariable in green and absent genes in white (Source: KEGG Mapper tool) (A). The hierarchical clusterization heatmap for the large (B) and small (C) subunits. The y-axis shows gene codes and x-axis shows female (F) and male (M) time courses from 2 to 96 hours. B3 is a cluster name described in Fig 1. Up-regulated genes are highlighted by red arrows for females.
(TIF)

S7 Fig Ribosome Biogenesis Pathway expression profile. Hierarchical clusterization of the ribosome biogenesis pathway in the post-emergence time points of 2, 12, 24, 48, and 96 hours. Heatmap y-axis shows gene codes and x-axis the female (F) and male (M) time courses from 2 to 96 hours. B3 is a cluster name described in Fig 1. Up-regulated genes are highlighted by red arrows for females.
(TIF)

S8 Fig Enriched Gene Ontology (GO) categories for the differentially expressed genes (DEGs). Enrichment analysis was performed with Panther scoring tool. The y-axis shows the GOs enriched for each ontology, as the x-axis the number of genes for the body (**A**) and the head (**B**).
(TIF)

S9 Fig DNA replication pathway, genes that regulate the cell cycle progression, apoptosis and histones. Hierarchical clusterization heatmap for the DNA replication pathway **(A)** and body differentially expressed genes (DEGs) for cell cycle progression, apoptosis and histones **(B)**. The y-axis shows gene codes and x-axis shows female (F) and male (M) time courses from 2 to 96 hours. B2 and B3 are cluster names described in Fig 1. Up-regulated genes are highlighted by red arrows for females.
(TIF)

S10 Fig DNA mismatch repair pathway expression profile. The hierarchical clusterization heatmap y-axis shows gene codes and x-axis shows female (F) and male (M) time courses from 2 to 96 hours. B3 is a cluster name described in Fig 1. Up-regulated genes are highlighted by red arrows for females **(A)**. Differentially expressed genes (DEG) names are in red boxes (up-regulated in female body—group B3), genes not DEG but with similar expression profile in pink, dissimilar or invariable in green and absent genes in white (Source: KEGG Mapper tool) **(B)**.
(TIF)

S11 Fig Muscle development and movement. The hierarchical clusterization heatmap y-axis shows gene codes and x-axis shows female (F) and male (M) time courses from 2 to 96 hours. H1 and H2 are cluster names described in Fig 1. Up-regulated genes are highlighted by red arrows for females.
(TIF)

S12 Fig Oxidative phosphorylation pathway. The hierarchical clusterization heatmap y-axis shows gene codes and x-axis shows female (F) and male (M) time courses from 2 to 96 hours. H2 is a cluster name described in Fig 1. Up-regulated genes are highlighted by red arrows for females.
(TIF)

S13 Fig Electron transport chain and normalized oxygen consumption rates (OCR). The mitochondrial pyruvate carrier (MPC 1 and MPC2) transports the pyruvate across the mitochondrial inner membrane into the mitochondrial matrix. The pyruvate dehydrogenase complex is composed by pyruvate dehydrogenase A (PDHA) and B (PDHB), dihydrolipoyllysine-residue acetyltransferase oxydase (DLAT) and dihydrolipoyl dehydrogenase (DLD). This complex mediates de oxidative decarboxylation of pyruvate to acetyl-CoA, producing CO_2_ and NADH. Proline is a main energy source for ATP synthesis in insects through OXPHOS. Although the molecular nature of mitochondrial proline transporter remains unknown, the first step of proline metabolism is mediated by proline desidrogenase (ProDH), generating 1-pyrroline-5-carboxylate (Δ1PC) and ubiquinol. Δ1PC is then oxidised to a-ketoglutarate (a-KG), producing NADH and glutamate. The expression profile of the MPCs and the pyruvate dehydrogenase complex can be observed in Fig 5A. Blunted arrows indicate the inhibitors used for each complex in our experiments **(A)**. The head OCRs were normalized by CS activity. Oxygen consumption coupled with oxidative phosphorylation (OXPHOS) **(B)**; maximum respiratory rates (ETS) **(C)**; Cytochrome c oxidase activity **(D)**; Leak represents the oxygen consumption in the presence of high substrate concentration but in the absence of ADP **(E)**; residual oxygen consumption (ROX) **(F)**. Bar graphs show mean (SEM) for males and females at 2, 12, 24, 48, and 96h post-emergence. Interaction p-value between sex and time factors (two-way ANOVA) were not significant (p>0.05) for all panels.
(TIF)

S14 Fig Mitochondrial biogenesis related genes. The hierarchical clusterization heatmap y-axis shows gene codes and x-axis shows female (F) and male (M) time courses from 2 to 96 hours.
(TIF)

S1 Table Summary of the sequencing performance across 72 samples represented by the number of reads generated (Raw reads), the number of reads that have quality enough to be analysed (Cleaned reads), the number of reads actually mapped against *Ae*. *aegypti* (version 5.1) transcripts (Mapped reads), the percentage of reads that actually mapped against *Ae*. *aegypti* (version 5.1) transcripts (% Mapped reads); the number of transcripts (*Ae*. *aegypti* version 5.1) that actually had mapped reads. (XLSX)

S2 Table Differentially expressed genes (DEGs) of head (A) and body (B) of Aedes Rio mosquitos along the first hours post-emergence (PE).
(XLSX)

S3 Table Primers used for qPCR. (XLSX)

S4 Table Numbers used in figures. (XLSX)

## References

[1] Takken W, Verhulst NO. Host preferences of blood-feeding mosquitoes. Annu Rev Entomol. 2013;58: 433–453. 10.1146/annurev-ento-120811-153618 23020619

[2] Hansen IA, Attardo GM, Park J-H, Peng Q, Raikhel AS. Target of rapamycin-mediated amino acid signaling in mosquito anautogeny. Proc Natl Acad Sci U S A. 2004;101: 10626–10631. 10.1073/pnas.0403460101 15229322 PMC489984

[3] Beklemishev WN. Gonotrophic rhythm as a basic principle of the biology of Anopheles. Vopr.Fiziol. Ekol. Malar. Komara. 1940;1: 3–22.http://paperpile.com/b/x4BkJW/53Fli

[4] Foster WA. Mosquito sugar feeding and reproductive energetics. Annu Rev Entomol. 1995;40: 443–474. 10.1146/annurev.en.40.010195.002303 7810991

[5] Klowden MJ. Endocrine aspects of mosquito reproduction. Archives of Insect Biochemistry and Physiology. 1997 pp. 491–512. 10.1002/(sici)1520-6327(1997)35:4<491::aid-arch10>3.3.co;2-5

[6] Hernández-Martínez S, Rivera-Perez C, Nouzova M, Noriega FG. Coordinated changes in JH biosynthesis and JH hemolymph titers in Aedes aegypti mosquitoes. Journal of Insect Physiology. 2015 pp. 22–27. 10.1016/j.jinsphys.2014.11.003 25445664 PMC4333059

[7] Ashok M, Turner C, Wilson TG. Insect juvenile hormone resistance gene homology with the bHLH-PAS family of transcriptional regulators. Proc Natl Acad Sci U S A. 1998;95: 2761–2766. 10.1073/pnas.95.6.2761 9501163 PMC19642

[8] Li M, Mead EA, Zhu J. Heterodimer of two bHLH-PAS proteins mediates juvenile hormone-induced gene expression. Proc Natl Acad Sci U S A. 2011;108: 638–643. 10.1073/pnas.1013914108 21187375 PMC3021087

[9] Li M, Liu P, Wiley JD, Ojani R, Bevan DR, Li J, et al A steroid receptor coactivator acts as the DNA-binding partner of the methoprene-tolerant protein in regulating juvenile hormone response genes. Mol Cell Endocrinol. 2014;394: 47–58. 10.1016/j.mce.2014.06.021 25004255 PMC4163509

[10] Zhang Z, Xu J, Sheng Z, Sui Y, Palli SR. Steroid receptor co-activator is required for juvenile hormone signal transduction through a bHLH-PAS transcription factor, methoprene tolerant. J Biol Chem. 2011;286: 8437–8447. 10.1074/jbc.M110.191684 21190938 PMC3048728

[11] Raikhel AS, Lea AO. Previtellogenic development and vitellogenin synthesis in the fat body of a mosquito: an ultrastructural and immunocytochemical study. Tissue Cell. 1983;15: 281–299. 10.1016/0040-8166(83)90023-x 6349013

[12] Wang J-L, Saha TT, Zhang Y, Zhang C, Raikhel AS. Juvenile hormone and its receptor methoprene-tolerant promote ribosomal biogenesis and vitellogenesis in the mosquito. J Biol Chem. 2017;292: 10306–10315. 10.1074/jbc.M116.761387 28446607 PMC5473233

[13] Raikhel AS, Lea AO. Hormone-mediated formation of the endocytic complex in mosquito oocytes. Gen Comp Endocrinol. 1985;57: 422–433. 10.1016/0016-6480(85)90224-2 3988025

[14] Gwadz RW, Spielman A. Corpus allatum control of ovarian development in Aedes aegypti. J Insect Physiol. 1973;19: 1441–1448. 10.1016/0022-1910(73)90174-1 4720505

[15] Zou Z, Saha TT, Roy S, Shin SW, Backman TWH, Girke T, et al Juvenile hormone and its receptor, methoprene-tolerant, control the dynamics of mosquito gene expression. Proc Natl Acad Sci U S A. 2013;110: E2173–81. 10.1073/pnas.1305293110 23633570 PMC3683779

[16] Zou Z, Saha TT, Roy S, Shin SW, Backman TWH, Girke T, et al Juvenile hormone and its receptor, methoprene-tolerant, control the dynamics of mosquito gene expression. Proc Natl Acad Sci U S A. 2013;110: E2173–81. 10.1073/pnas.1305293110 23633570 PMC3683779

[17] Reid WR, Zhang L, Liu N. Temporal Gene Expression Profiles of Pre Blood-Fed Adult Females Immediately Following Eclosion in the Southern House Mosquito Culex Quinquefasciatus. Int J Biol Sci. 2015;11: 1306–1313. 10.7150/ijbs.12829 26435696 PMC4582154

[18] Hou Y, Wang X-L, Saha TT, Roy S, Zhao B, Raikhel AS, et al Temporal Coordination of Carbohydrate Metabolism during Mosquito Reproduction. PLoS Genet. 2015;11: e1005309 10.1371/journal.pgen.1005309 26158648 PMC4497655

[19] Fernandes KM, de Magalhães-Júnior MJ, Baracat-Pereira MC, Martins GF. Proteomic analysis of Aedes aegypti midgut during post-embryonic development and of the female mosquitoes fed different diets. Parasitol Int. 2016;65: 668–676. 10.1016/j.parint.2016.08.008 27597118

[20] Ribeiro JMC, Martin-Martin I, Arcà B, Calvo E. A Deep Insight into the Sialome of Male and Female Aedes aegypti Mosquitoes. PLoS One. 2016;11: e0151400 10.1371/journal.pone.0151400 26999592 PMC4801386

[21] Tallon AK, Hill SR, Ignell R. Sex and age modulate antennal chemosensory-related genes linked to the onset of host seeking in the yellow-fever mosquito, Aedes aegypti. Sci Rep. 2019;9: 43 10.1038/s41598-018-36550-6 30631085 PMC6328577

[22] Santos CR dos, dos Santos CR, de Melo Rodovalho C, Jablonka W, Martins AJ, Lima JBP, et al Insecticide resistance, fitness and susceptibility to Zika infection of an interbred Aedes aegypti population from Rio de Janeiro, Brazil. Parasites & Vectors. 2020 10.1186/s13071-020-04166-3 32513248 PMC7281914

[23] DeGennaro M, McBride CS, Seeholzer L, Nakagawa T, Dennis EJ, Goldman C, et al orco mutant mosquitoes lose strong preference for humans and are not repelled by volatile DEET. Nature. 2013 pp. 487–491. 10.1038/nature12206 23719379 PMC3696029

[24] Lowry OH, Rosebrough NJ, Farr AL, Randall RJ. Protein measurement with the Folin phenol reagent. J Biol Chem. 1951;193: 265–275. 14907713

[25] Andrews S. et al FastQC. A quality control tool for high throughput sequence data. Babraham Bioinformatics. 2015; 1:1.

[26] Martin M. Cutadapt removes adapter sequences from high-throughput sequencing reads. EMBnet.journal. 2011 p. 10 10.14806/ej.17.1.200

[27] Langmead B, Salzberg SL. Fast gapped-read alignment with Bowtie 2. Nat Methods. 2012;9: 357–359. 10.1038/nmeth.1923 22388286 PMC3322381

[28] Patro R, Duggal G, Love MI, Irizarry RA, Kingsford C. Salmon provides fast and bias-aware quantification of transcript expression. Nat Methods. 2017;14: 417–419. 10.1038/nmeth.4197 28263959 PMC5600148

[29] Zhang C, Zhang B, Lin L-L, Zhao S. Evaluation and comparison of computational tools for RNA-seq isoform quantification. BMC Genomics. 2017;18: 583 10.1186/s12864-017-4002-1 28784092 PMC5547501

[30] Love MI, Huber W, Anders S. Moderated estimation of fold change and dispersion for RNA-seq data with DESeq2. Genome Biol. 2014;15: 550 10.1186/s13059-014-0550-8 25516281 PMC4302049

[31] Shamir R, Maron-Katz A, Tanay A, Linhart C, Steinfeld I, Sharan R, et al EXPANDER—an integrative program suite for microarray data analysis. BMC Bioinformatics. 2005;6: 232 10.1186/1471-2105-6-232 16176576 PMC1261157

[32] Mi H, Dong Q, Muruganujan A, Gaudet P, Lewis S, Thomas PD. PANTHER version 7: improved phylogenetic trees, orthologs and collaboration with the Gene Ontology Consortium. Nucleic Acids Res. 2010;38: D204–10. 10.1093/nar/gkp1019 20015972 PMC2808919

[33] Kanehisa M, Sato Y, Furumichi M, Morishima K, Tanabe M. New approach for understanding genome variations in KEGG. Nucleic Acids Res. 2019;47: D590–D595. 10.1093/nar/gky962 30321428 PMC6324070

[34] Kanehisa M, Furumichi M, Tanabe M, Sato Y, Morishima K. KEGG: new perspectives on genomes, pathways, diseases and drugs. Nucleic Acids Res. 2017;45: D353–D361. 10.1093/nar/gkw1092 27899662 PMC5210567

[35] Ogata H, Goto S, Sato K, Fujibuchi W, Bono H, Kanehisa M. KEGG: Kyoto Encyclopedia of Genes and Genomes. Nucleic Acids Res. 1999;27: 29–34. 10.1093/nar/27.1.29 9847135 PMC148090

[36] Li Y, Zhang L, Li R, Zhang M, Li Y, Wang H, et al Systematic identification and validation of the reference genes from 60 RNA-Seq libraries in the scallop Mizuhopecten yessoensis. BMC Genomics. 2019;20: 288 10.1186/s12864-019-5661-x 30975074 PMC6460854

[37] Bustin SA, Benes V, Garson JA, Hellemans J, Huggett J, Kubista M, et al The MIQE Guidelines: Minimum Information for Publication of Quantitative Real-Time PCR Experiments. Clinical Chemistry. 2009 pp. 611–622. 10.1373/clinchem.2008.112797 19246619

[38] Gaviraghi A, Oliveira MF. A method for assessing mitochondrial physiology using mechanically permeabilized flight muscle of Aedes aegypti mosquitoes. Anal Biochem. 2019;576: 33–41. 10.1016/j.ab.2019.04.005 30974092

[39] Gammone MA, Efthymakis K, Pluchinotta FR, Bergante S, Tettamanti G, Riccioni G, et al Impact of chocolate on the cardiovascular health. Front Biosci. 2018;23: 852–864. 10.2741/4620 28930576

[40] Soares JBRC, Gaviraghi A, Oliveira MF. Mitochondrial physiology in the major arbovirus vector Aedes aegypti: substrate preferences and sexual differences define respiratory capacity and superoxide production. PLoS One. 2015;10: e0120600 10.1371/journal.pone.0120600 25803027 PMC4372595

[41] Pesta D, Gnaiger E. High-resolution respirometry: OXPHOS protocols for human cells and permeabilized fibers from small biopsies of human muscle. Methods Mol Biol. 2012;810: 25–58. 10.1007/978-1-61779-382-0_3 22057559

[42] Chandel NS, Budinger GR, Schumacker PT. Molecular oxygen modulates cytochrome c oxidase function. J Biol Chem. 1996;271: 18672–18677. 10.1074/jbc.271.31.18672 8702521

[43] Srere PA, Brazil H, Gonen L, Takahashi M. The Citrate Condensing Enzyme of Pigeon Breast Muscle and Moth Flight Muscle. Acta Chemica Scandinavica. 1963 pp. 129–134. 10.3891/acta.chem.scand.17s-0129

[44] Christophers SR. Aedes Aegypti the yellow fever mosquito: Its life history, bionomics ans structure. Cambridge University Press 1960; pp. 752.

[45] Champagne DE, Ribeiro JM. Sialokinin I and II: vasodilatory tachykinins from the yellow fever mosquito Aedes aegypti. Proc Natl Acad Sci U S A. 1994;91: 138–142. 10.1073/pnas.91.1.138 8278354 PMC42901

[46] Champagne DE, Smartt CT, Ribeiro JM, James AA. The salivary gland-specific apyrase of the mosquito Aedes aegypti is a member of the 5’-nucleotidase family. Proc Natl Acad Sci U S A. 1995;92: 694–698. 10.1073/pnas.92.3.694 7846038 PMC42686

[47] Stark KR, James AA. Isolation and characterization of the gene encoding a novel factor Xa-directed anticoagulant from the yellow fever mosquito, Aedes aegypti. J Biol Chem. 1998;273: 20802–20809. 10.1074/jbc.273.33.20802 9694825

[48] Chagas AC, Ramirez JL, Jasinskiene N, James AA, Ribeiro JMC, Marinotti O, et al Collagen-binding protein, Aegyptin, regulates probing time and blood feeding success in the dengue vector mosquito, Aedes aegypti. Proc Natl Acad Sci U S A. 2014;111: 6946–6951. 10.1073/pnas.1404179111 24778255 PMC4024861

[49] Ribeiro JMC, Arcà B, Lombardo F, Calvo E, Phan VM, Chandra PK, et al An annotated catalogue of salivary gland transcripts in the adult female mosquito, Aedes aegypti. BMC Genomics. 2007;8: 6 10.1186/1471-2164-8-6 17204158 PMC1790711

[50] Rossignol PA, Lueders AM. Bacteriolytic factor in the salivary glands of Aedes aegypti. Comp Biochem Physiol B. 1986;83: 819–822. 10.1016/0305-0491(86)90153-7 3519067

[51] Noriega FG, Edgar KA, Bechet R, Wells MA. Midgut exopeptidase activities in Aedes aegypti are induced by blood feeding. J Insect Physiol. 2002;48: 205–212. 10.1016/s0022-1910(01)00165-2 12770120

[52] Isoe J, Rascón AA Jr, Kunz S, Miesfeld RL. Molecular genetic analysis of midgut serine proteases in Aedes aegypti mosquitoes. Insect Biochem Mol Biol. 2009;39: 903–912. 10.1016/j.ibmb.2009.10.008 19883761 PMC2818436

[53] Brackney DE, Isoe J, W C B 4th, Zamora J, Foy BD, Miesfeld RL, et al Expression profiling and comparative analyses of seven midgut serine proteases from the yellow fever mosquito, Aedes aegypti. J Insect Physiol. 2010;56: 736–744. 10.1016/j.jinsphys.2010.01.003 20100490 PMC2878907

[54] Bian G, Raikhel AS, Zhu J. Characterization of a juvenile hormone-regulated chymotrypsin-like serine protease gene in Aedes aegypti mosquito. Insect Biochem Mol Biol. 2008;38: 190–200. 10.1016/j.ibmb.2007.10.008 18207080 PMC2253661

[55] Jiang Q, Hall M, Noriega FG, Wells M. cDNA cloning and pattern of expression of an adult, female-specific chymotrypsin from Aedes aegypti midgut. Insect Biochem Mol Biol. 1997;27: 283–289. 10.1016/s0965-1748(97)00001-5 9134710

[56] Kalhok SE, Tabak LM, Prosser DE, Brook W, Downe AE, White BN. Isolation, sequencing and characterization of two cDNA clones coding for trypsin-like enzymes from the midgut of Aedes aegypti. Insect Mol Biol. 1993;2: 71–79. 10.1111/j.1365-2583.1993.tb00127.x 9087545

[57] Noriega FG, Pennington JE, Barillas-Mury C, Wang XY, Wells MA. Aedes aegypti midgut early trypsin is post-transcriptionally regulated by blood feeding. Insect Molecular Biology. 1996 pp. 25–29. 10.1111/j.1365-2583.1996.tb00037.x 8630532

[58] Barillas-Mury C, Wells MA. Cloning and sequencing of the blood meal-induced late trypsin gene from the mosquito Aedes aegypti and characterization of the upstream regulatory region. Insect Mol Biol. 1993;2: 7–12. 10.1111/j.1365-2583.1993.tb00119.x 9087537

[59] Dong S, Behura SK, Franz AWE. The midgut transcriptome of Aedes aegypti fed with saline or protein meals containing chikungunya virus reveals genes potentially involved in viral midgut escape. BMC Genomics. 2017;18: 382 10.1186/s12864-017-3775-6 28506207 PMC5433025

[60] Saha TT, Shin SW, Dou W, Roy S, Zhao B, Hou Y, et al Hairy and Groucho mediate the action of juvenile hormone receptor Methoprene-tolerant in gene repression. Proc Natl Acad Sci U S A. 2016;113: E735–43. 10.1073/pnas.1523838113 26744312 PMC4760797

[61] Saha TT, Roy S, Pei G, Dou W, Zou Z, Raikhel AS. Synergistic action of the transcription factors Krüppel homolog 1 and Hairy in juvenile hormone/Methoprene-tolerant-mediated gene-repression in the mosquito Aedes aegypti. PLoS Genet. 2019;15: e1008443 10.1371/journal.pgen.1008443 31661489 PMC6818763

[62] Raikhel AS, Lea AO. Juvenile hormone controls previtellogenic proliferation of ribosomal RNA in the mosquito fat body. Gen Comp Endocrinol. 1990;77: 423–434. 10.1016/0016-6480(90)90233-c 1970970

[63] Raikhel A. Accumulation Of Yolk Proteins In Insect Oocytes. Annual Review of Entomology. 1992 pp. 217–251. 10.1146/annurev.en.37.010192.001245 1311540

[64] Clifton ME, Noriega FG. The fate of follicles after a blood meal is dependent on previtellogenic nutrition and juvenile hormone in Aedes aegypti. J Insect Physiol. 2012;58: 1007–1019. 10.1016/j.jinsphys.2012.05.005 22626792 PMC3389259

[65] Fox DT, Duronio RJ. Endoreplication and polyploidy: insights into development and disease. Development. 2013;140: 3–12. 10.1242/dev.080531 23222436 PMC3513989

[66] Lilly MA, Duronio RJ. New insights into cell cycle control from the Drosophila endocycle. Oncogene. 2005 pp. 2765–2775. 10.1038/sj.onc.1208610 15838513

[67] van den Heuvel S, Dyson NJ. Conserved functions of the pRB and E2F families. Nat Rev Mol Cell Biol. 2008;9: 713–724. 10.1038/nrm2469 18719710

[68] Liu D, Keijzers G, Rasmussen LJ. DNA mismatch repair and its many roles in eukaryotic cells. Mutat Res. 2017;773: 174–187. 10.1016/j.mrrev.2017.07.001 28927527

[69] Mota MBS, Carvalho MA, Monteiro ANA, Mesquita RD. DNA damage response and repair in perspective: Aedes aegypti, Drosophila melanogaster and Homo sapiens. Parasit Vectors. 2019;12: 533 10.1186/s13071-019-3792-1 31711518 PMC6849265

[70] Larsen S, Nielsen J, Hansen CN, Nielsen LB, Wibrand F, Stride N, et al Biomarkers of mitochondrial content in skeletal muscle of healthy young human subjects. J Physiol. 2012;590: 3349–3360. 10.1113/jphysiol.2012.230185 22586215 PMC3459047

[71] Andersson U, Scarpulla RC. PGC-1-Related Coactivator, a Novel, Serum-Inducible Coactivator of Nuclear Respiratory Factor 1-Dependent Transcription in Mammalian Cells. Molecular and Cellular Biology. 2001 pp. 3738–3749. 10.1128/MCB.21.11.3738-3749.2001 11340167 PMC87014

[72] Scarpulla RC. Nuclear Control of Respiratory Chain Expression by Nuclear Respiratory Factors and PGC-1-Related Coactivator. Annals of the New York Academy of Sciences. 2008 pp. 321–334. 10.1196/annals.1427.006 19076454 PMC2853241

[73] Handschin C, Lin J, Rhee J, Peyer A-K, Chin S, Wu P-H, et al Nutritional regulation of hepatic heme biosynthesis and porphyria through PGC-1alpha. Cell. 2005;122: 505–515. 10.1016/j.cell.2005.06.040 16122419

[74] Wang X, Hou Y, Saha TT, Pei G, Raikhel AS, Zou Z. Hormone and receptor interplay in the regulation of mosquito lipid metabolism. Proc Natl Acad Sci U S A. 2017;114: E2709–E2718. 10.1073/pnas.1619326114 28292900 PMC5380040

[75] Davis EE. Development of lactic acid-receptor sensitivity and host-seeking behaviour in newly emerged female Aedes aegypti mosquitoes. Journal of Insect Physiology. 1984 pp. 211–215. 10.1016/0022-1910(84)90005-2

[76] Graf R, Raikhel AS, Brown MR, Lea AO, Briegel H. Mosquito trypsin: immunocytochemical localization in the midgut of blood-fed Aedes aegypti (L.). Cell Tissue Res. 1986;245: 19–27. 10.1007/BF00218082 3524850

[77] Barillas-Mury C, Graf R, Hagedorn HH, Wells MA. cDNA and deduced amino acid sequence of a blood meal-induced trypsin from the mosquito, Aedes aegypti. Insect Biochemistry. 1991 pp. 825–831. 10.1016/0020-1790(91)90089-w

[78] Noriega FG, Wells MA. A molecular view of trypsin synthesis in the midgut of Aedes aegypti. J Insect Physiol. 1999;45: 613–620. 10.1016/s0022-1910(99)00052-9 12770346

[79] Noriega FG, Shah DK, Wells MA. Juvenile hormone controls early trypsin gene transcription in the midgut of Aedes aegypti. Insect Mol Biol. 1997;6: 63–66. 10.1046/j.1365-2583.1997.00154.x 9013256

[80] Graf R, Binz H, Briegel H. Monoclonal antibodies as probes for Aedes aegypti trypsin. Insect Biochemistry. 1988 pp. 463–470. 10.1016/0020-1790(88)90063-7

[81] Hancock RG, Foster WA. Exogenous Juvenile Hormone and methoprene, but not male accessory gland substances or ovariectomy, affect the blood/nectar choice of female Culex nigripalpus mosquitoes. Medical and Veterinary Entomology. 2000 pp. 376–382. 10.1046/j.1365-2915.2000.00253.x 11129701

[82] Jindra M, Palli SR, Riddiford LM. The Juvenile Hormone Signaling Pathway in Insect Development. Annual Review of Entomology. 2013 pp. 181–204. 10.1146/annurev-ento-120811-153700 22994547

[83] Santos CG, Humann FC, Hartfelder K. Juvenile hormone signaling in insect oogenesis. Curr Opin Insect Sci. 2019;31: 43–48. 10.1016/j.cois.2018.07.010 31109672

[84] Cho KH, Raikhel AS. Organization and developmental expression of the mosquito vitellogenin receptor gene. Insect Mol Biol. 2001;10: 465–474. 10.1046/j.0962-1075.2001.00285.x 11881811

[85] Øvrebø JI, Edgar BA. Polyploidy in tissue homeostasis and regeneration. Development. 2018 p. dev156034. 10.1242/dev.156034 30021843 PMC10682953

[86] Wu Z, Guo W, Xie Y, Zhou S. Juvenile Hormone Activates the Transcription of Cell-division-cycle 6 (Cdc6) for Polyploidy-dependent Insect Vitellogenesis and Oogenesis. J Biol Chem. 2016;291: 5418–5427. 10.1074/jbc.M115.698936 26728459 PMC4777871

[87] Guo W, Wu Z, Song J, Jiang F, Wang Z, Deng S, et al Juvenile hormone-receptor complex acts on mcm4 and mcm7 to promote polyploidy and vitellogenesis in the migratory locust. PLoS Genet. 2014;10: e1004702 10.1371/journal.pgen.1004702 25340846 PMC4207617

[88] Xiang J, Bandura J, Zhang P, Jin Y, Reuter H, Edgar BA. EGFR-dependent TOR-independent endocycles support Drosophila gut epithelial regeneration. Nat Commun. 2017;8: 15125 10.1038/ncomms15125 28485389 PMC5436070

[89] Zielke N, Kim KJ, Tran V, Shibutani ST, Bravo M-J, Nagarajan S, et al Control of Drosophila endocycles by E2F and CRL4CDT2. Nature. 2011 pp. 123–127. 10.1038/nature10579 22037307 PMC3330263

[90] Wu Z, Guo W, Yang L, He Q, Zhou S. Juvenile hormone promotes locust fat body cell polyploidization and vitellogenesis by activating the transcription of Cdk6 and E2f1. Insect Biochem Mol Biol. 2018;102: 1–10. 10.1016/j.ibmb.2018.09.002 30205150

[91] Hassel C, Zhang B, Dixon M, Calvi BR. Induction of endocycles represses apoptosis independently of differentiation and predisposes cells to genome instability. Development. 2014;141: 112–123. 10.1242/dev.098871 24284207 PMC3865753

[92] Liu Q, Clem RJ. Defining the core apoptosis pathway in the mosquito disease vector Aedes aegypti: the roles of iap1, ark, dronc, and effector caspases. Apoptosis. 2011;16: 105–113. 10.1007/s10495-010-0558-9 21107703 PMC6029261

[93] Qiu F, Brendel S, Cunha PMF, Astola N, Song B, Furlong EEM, et al Myofilin, a protein in the thick filaments of insect muscle. J Cell Sci. 2005;118: 1527–1536. 10.1242/jcs.02281 15769842

[94] Vigoreaux JO, Hernandez C, Moore J, Ayer G, Maughan D. A genetic deficiency that spans the flightin gene of Drosophila melanogaster affects the ultrastructure and function of the flight muscles. J Exp Biol. 1998;201: 2033–2044. 9622575 10.1242/jeb.201.13.2033

[95] Reedy MC, Bullard B, Vigoreaux JO. Flightin is essential for thick filament assembly and sarcomere stability in Drosophila flight muscles. J Cell Biol. 2000;151: 1483–1500. 10.1083/jcb.151.7.1483 11134077 PMC2150682

[96] Henkin JA, Maughan DW, Vigoreaux JO. Mutations that affect flightin expression in Drosophila alter the viscoelastic properties of flight muscle fibers. Am J Physiol Cell Physiol. 2004;286: C65–72. 10.1152/ajpcell.00257.2003 12954604

[97] Lehman W, Craig R, Vibert P. Ca2 -induced tropomyosin movement in Limulus thin filaments revealed by three-dimensional reconstruction. Nature. 1994 pp. 65–67. 10.1038/368065a0 8107884

[98] Kikuchi K, Mochizuki O. Study of the Pump of a Mosquito. ASME-JSME-KSME 2011 Joint Fluids Engineering Conference: Volume 1, Symposia–Parts A, B, C, and D. 2011. 10.1115/ajk2011-19003

[99] Kim BH, Kim HK, Lee SJ. Experimental analysis of the blood-sucking mechanism of female mosquitoes. J Exp Biol. 2011;214: 1163–1169. 10.1242/jeb.048793 21389202

[100] Litonin D, Sologub M, Shi Y, Savkina M, Anikin M, Falkenberg M, et al Human Mitochondrial Transcription Revisited. Journal of Biological Chemistry. 2010 pp. 18129–18133. 10.1074/jbc.C110.128918 20410300 PMC2881736

[101] Laughlin SB, van Steveninck RR de R, Anderson JC. The metabolic cost of neural information. Nature Neuroscience. 1998 pp. 36–41. 10.1038/236 10195106

[102] Vallee R. Mechanisms Of Fast And Slow Axonal Transport. Annual Review of Neuroscience. 1991 pp. 59–92. 10.1146/annurev.ne.14.030191.000423 1709561

[103] Bindokas VP, Lee CC, Colmers WF, Miller RJ. Changes in mitochondrial function resulting from synaptic activity in the rat hippocampal slice. J Neurosci. 1998;18: 4570–4587. 10.1523/JNEUROSCI.18-12-04570.1998 9614233 PMC6792701

[104] Hollenbeck PJ. The pattern and mechanism of mitochondrial transport in axons. Front Biosci. 1996;1: d91–102. 10.2741/a118 9159217

[105] Goldstein LS, Yang Z. Microtubule-based transport systems in neurons: the roles of kinesins and dyneins. Annu Rev Neurosci. 2000;23: 39–71. 10.1146/annurev.neuro.23.1.39 10845058

[106] Górska-Andrzejak J, Stowers RS, Borycz J, Kostyleva R, Schwarz TL, Meinertzhagen IA. Mitochondria are redistributed in Drosophila photoreceptors lacking Milton, a kinesin-associated protein. Journal of Comparative Neurology. 2003 pp. 372–388. 10.1002/cne.10750 12836173

