# Supplementary figures and images for: Aedes aegypti post-emergence transcriptome: Unveiling the molecular basis for the hematophagic and gonotrophic capacitation
Source: PLoS Negl Trop Dis. 2021 Jan 6;15(1):e0008915. doi: 10.1371/journal.pntd.0008915 (PMC7815146; doi:10.1371/journal.pntd.0008915)

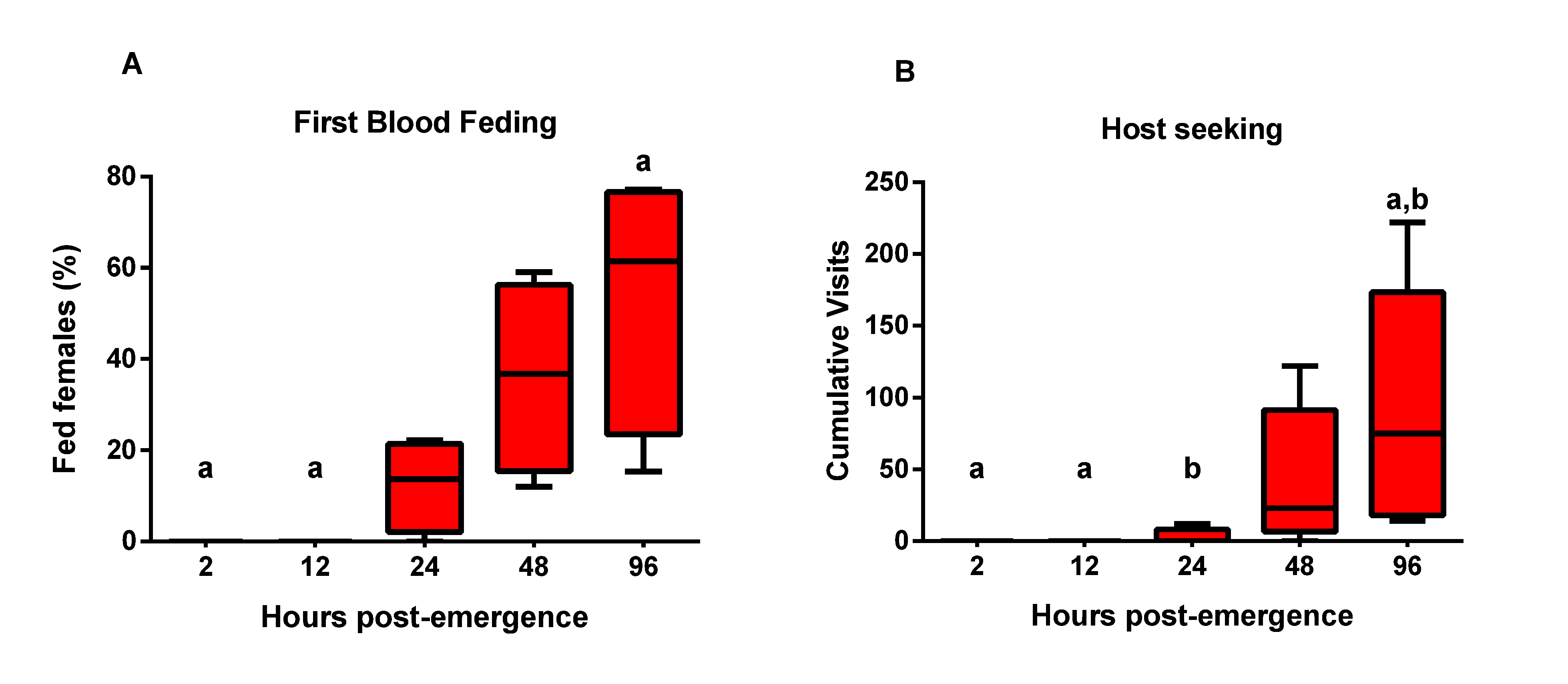

Supplement: S1 Fig — (A) Percent females freely feeding on an offered artificial blood meal; and (B) Cumulative number of female visits to the net in the area exposed to a human arm. The post-emergence time points tested were 2, 12, 24, 48, and 96 hours. Bars upper and lower whiskers represent the highest and lowest observations. The line inside the bar represents the median. (Kruskal-Wallis corrected one-way ANOVA comparing the hours post emergence; (a) P<0.005 (b) P<0.05). N = 5). (TIF) [file pntd.0008915.s001.tif]

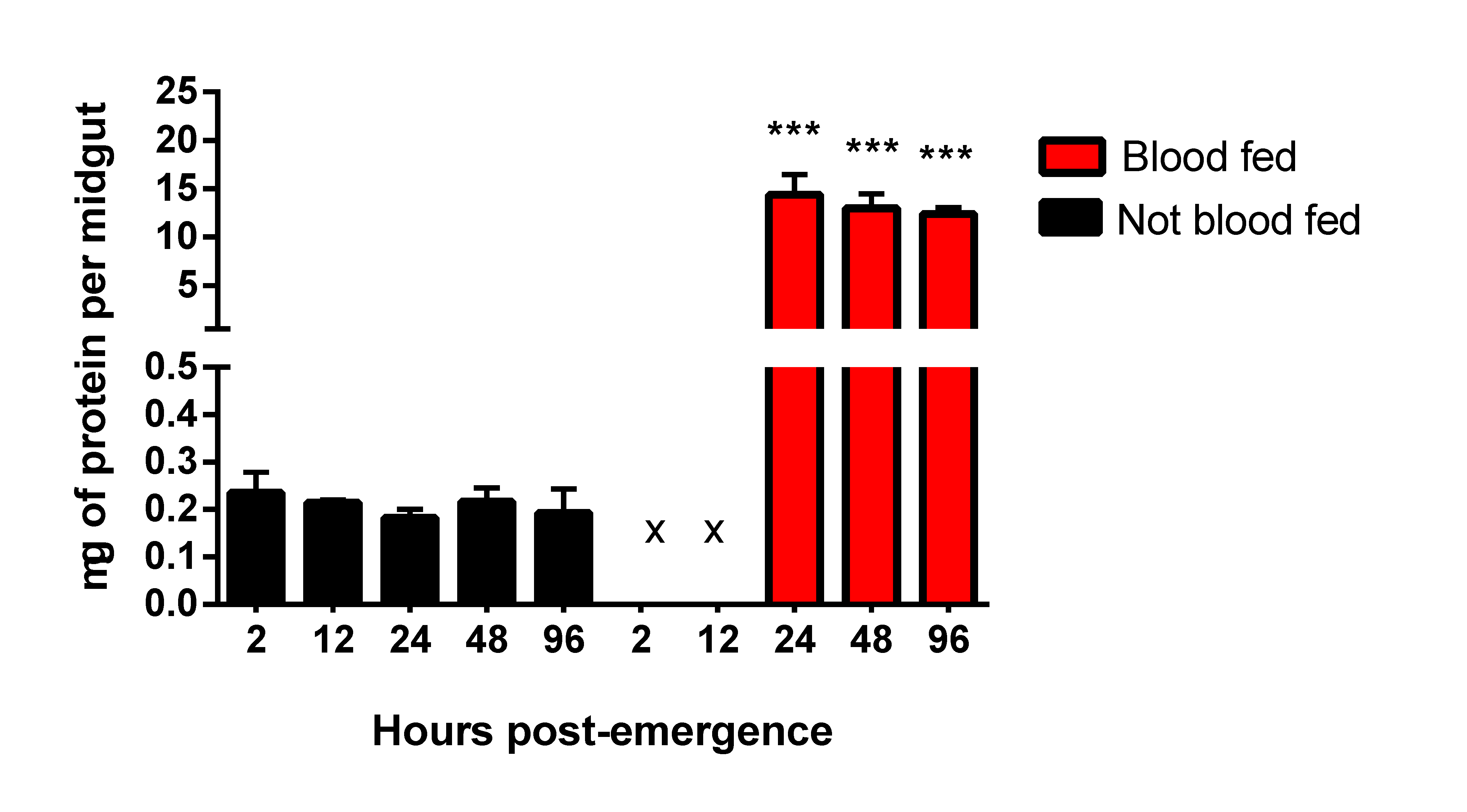

Supplement: S2 Fig — Total protein amount was quantified per midgut of fed and unfed females after offering an artificial blood meal for 30 minutes. "X", at 2 and 12h PE none mosquito took the blood meal. (Tukey’s corrected one-way ANOVA; *** P<0.0001. N = 4). (TIF) [file pntd.0008915.s002.tif]

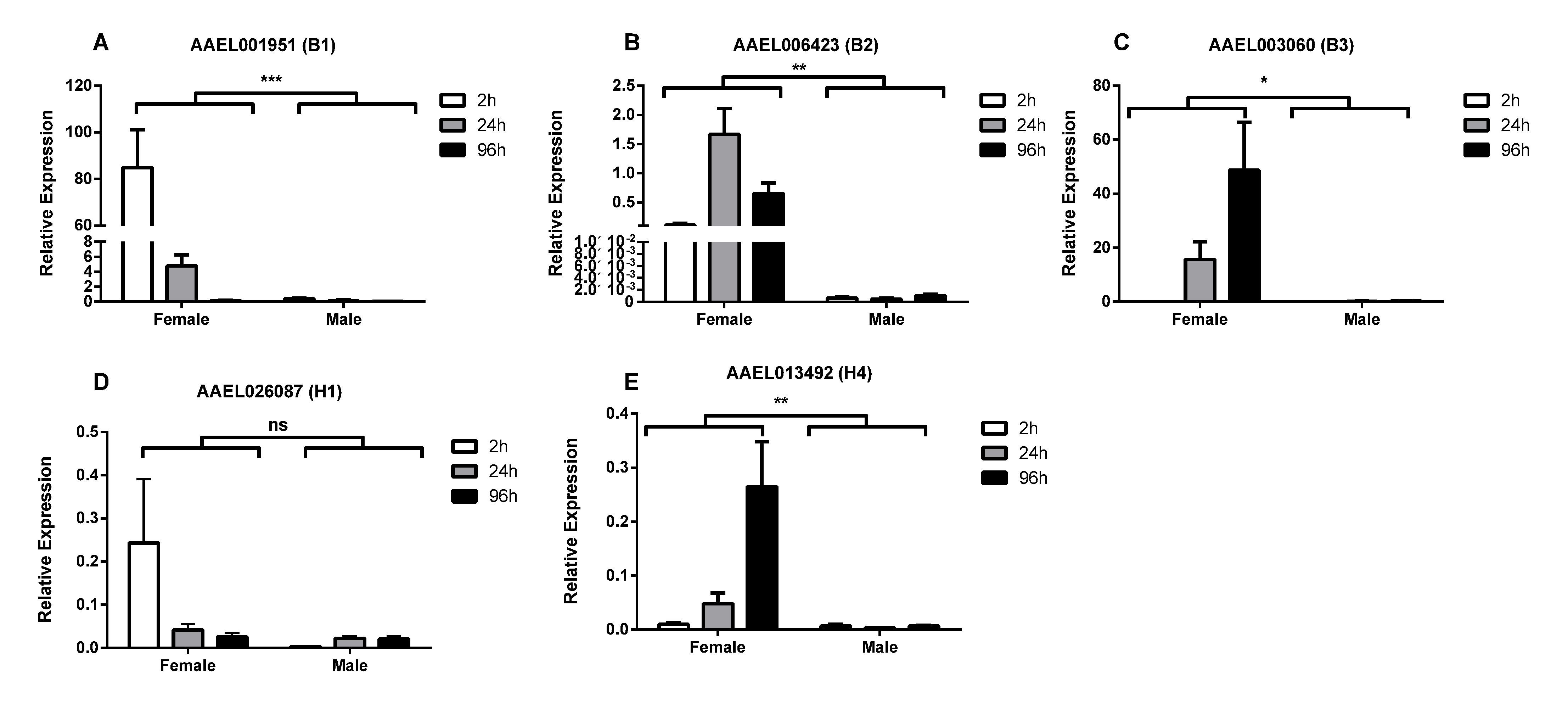

Supplement: S3 Fig — One gene was selected to represent the profile of clusters: B1 (A), B2 (B), B3 (C), H1 (D), and H4 (E). Bar graphs show mean (SEM) for males and females. The post-emergence time points tested were 2, 24, and 96 hours. Interaction p-values between sex and time factors (two-way ANOVA) are displayed above the bars. ns = not significant; *p-value <0.05; **p-value <0.01, ***p-value <0.001. (TIF) [file pntd.0008915.s003.tif]

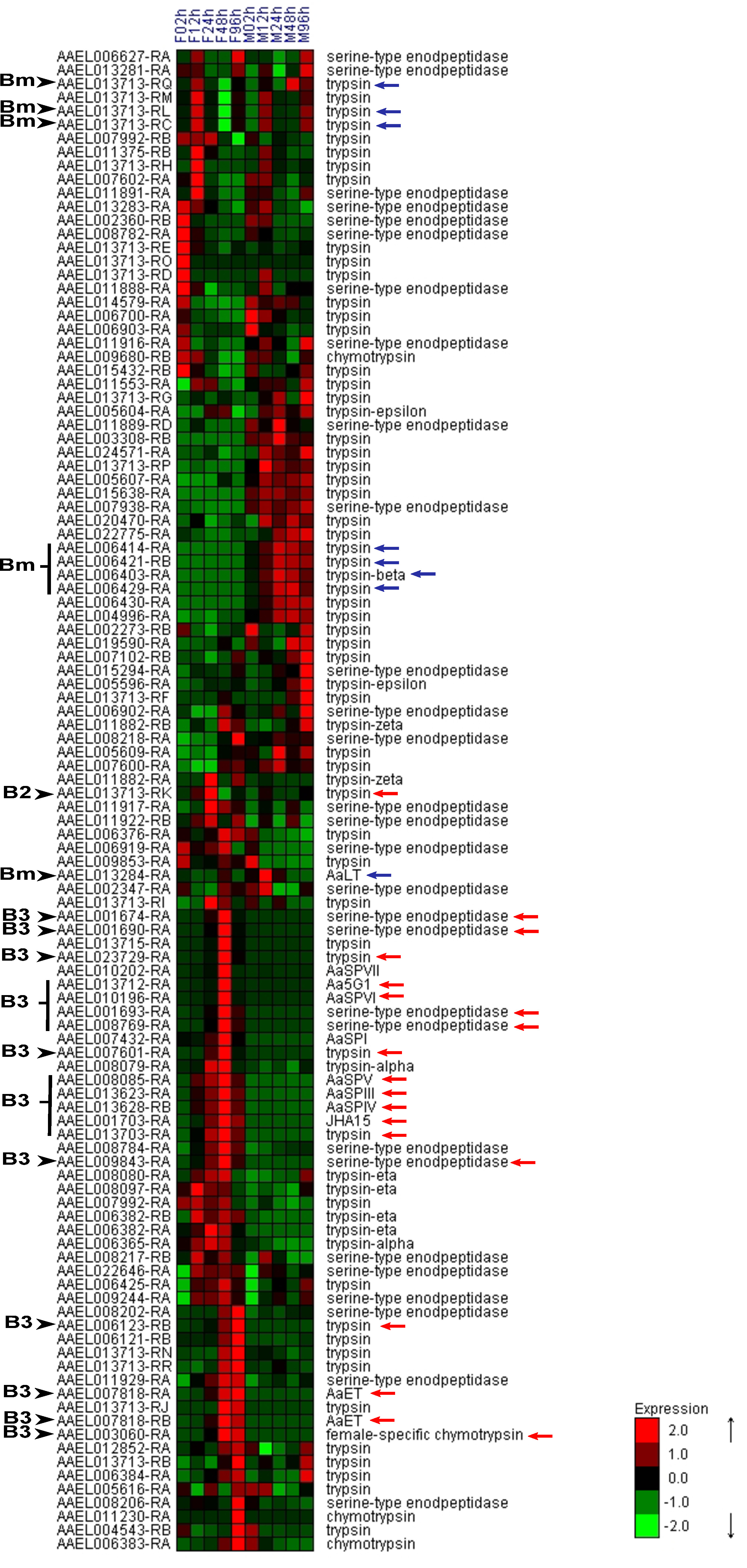

Supplement: S4 Fig — Hierarchical clusterization of the serine protease genes in the post-emergence time points of 2, 12, 24, 48, and 96 hours. Heatmap y-axis shows gene codes and x-axis the female (F) and male (M) time courses from 2 to 96 hours. B2, B3, and Bm are cluster names described in Fig 1. Up-regulated genes are highlighted by red arrows for females and blue arrows for males. (TIF) [file pntd.0008915.s004.tif]

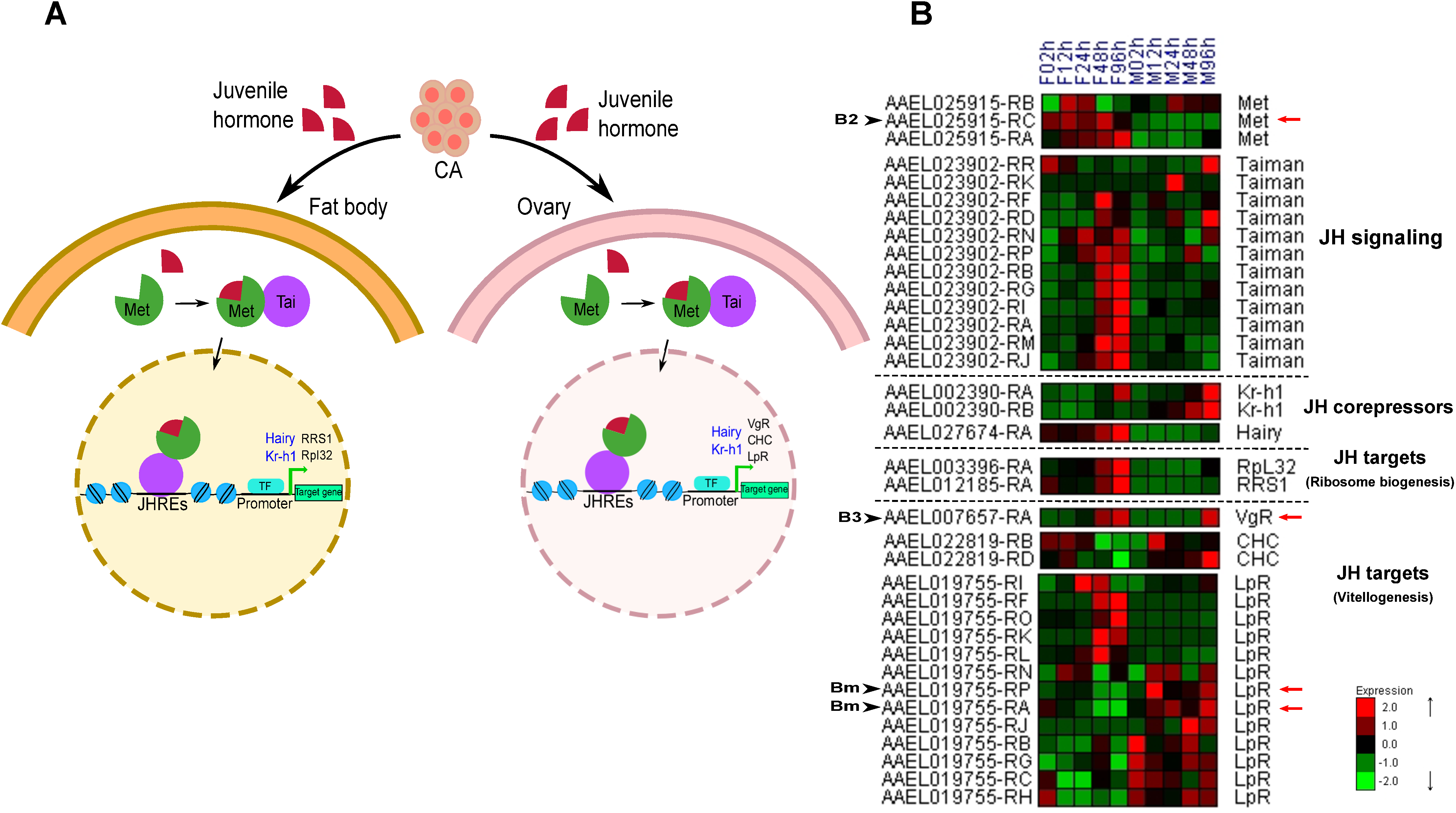

Supplement: S5 Fig — Summarized juvenile hormone (JH) signaling pathway (A) The JH biosynthesis occurs in the corpora allata (CA), a pair of endocrine glands with neural connections to the brain. There is a relevant JH increase in hemolymph 12h PE leading the fat body (FB) priming and maturation of oocytes for vitellogenesis. JH has an intracellular receptor Methoprene-tolerant (Met), which binds to Taiman (Tai), this heterodimer binds to Juvenile hormone response elements (JHREs) modulating gene expression. Hairy and Kruppel homolog 1 (Kr-h1) are transcription factors that will regulate the expression of other genes. In the FB, this pathway will activate Ribosome Biogenesis Regulator 1 Homolog (RRS1) and Ribosomal protein L32 (RpL32), required for Ribosome biogenesis pathway and subsequently the production of vitellogenin (Vg). In the ovary, the activation of this pathway will regulate the expression of genes such as vitellogenin receptor (VgR), Heavy-Chain Clathrin (CHC) and the lipophorin receptor (LpR), related to the uptake of vitellogenin (Vg); Heatmap of Juvenile hormone signaling pathway and the cited target genes for the body (B). Heatmap y-axis shows gene codes and x-axis shows female (F) and male (M) time courses from 2 to 96 hours. B2, B3, and Bm are cluster names described in Fig 1. Up-regulated genes are highlighted by red arrows for females. (TIF) [file pntd.0008915.s005.tif]

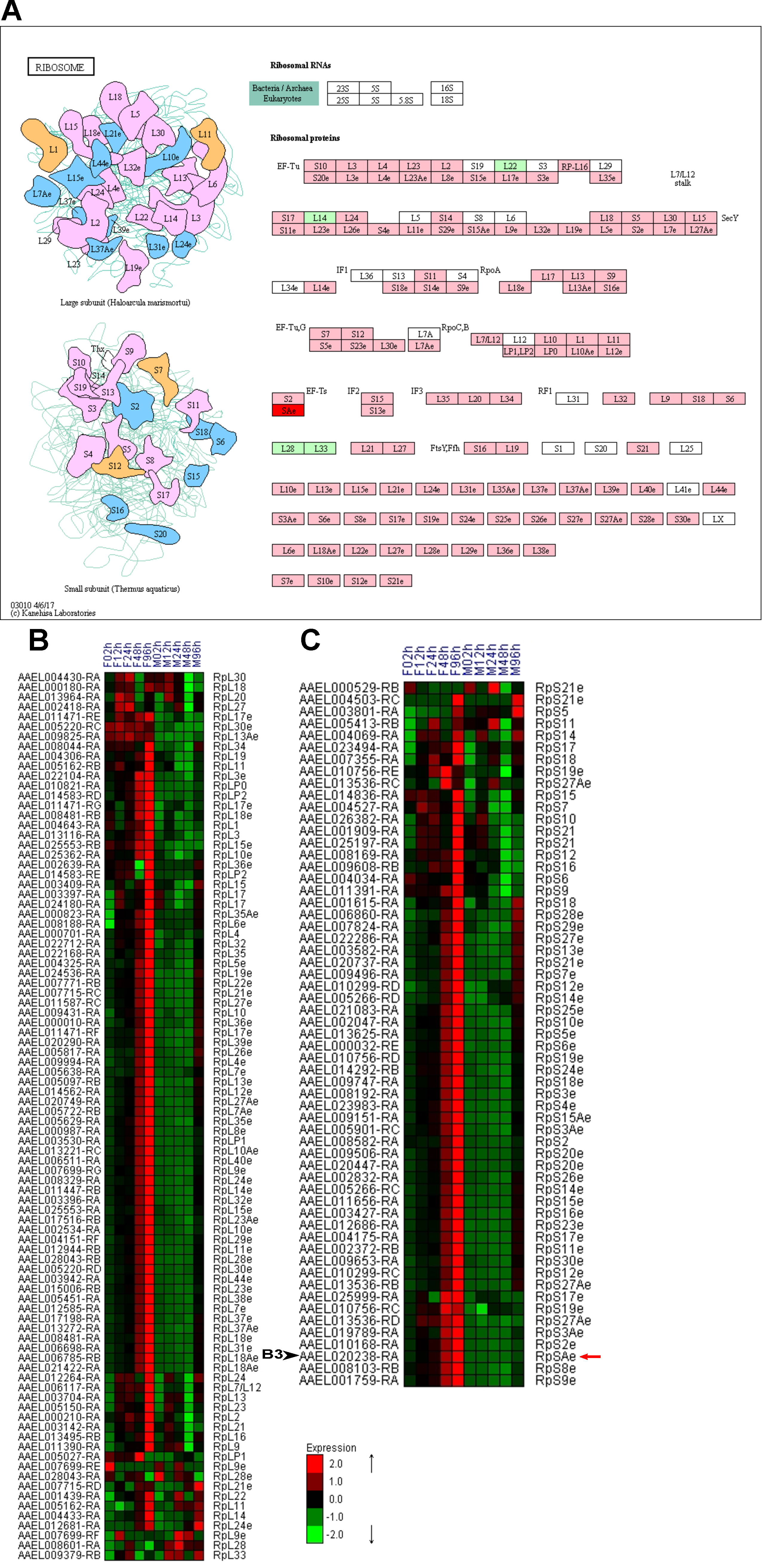

Supplement: S6 Fig — Differentially expressed genes (DEG) names are in red boxes (up-regulated in female body—group B3), genes not DEG but with similar expression profile in pink, dissimilar or invariable in green and absent genes in white (Source: KEGG Mapper tool) (A). The hierarchical clusterization heatmap for the large (B) and small (C) subunits. The y-axis shows gene codes and x-axis shows female (F) and male (M) time courses from 2 to 96 hours. B3 is a cluster name described in Fig 1. Up-regulated genes are highlighted by red arrows for females. (TIF) [file pntd.0008915.s006.tif]

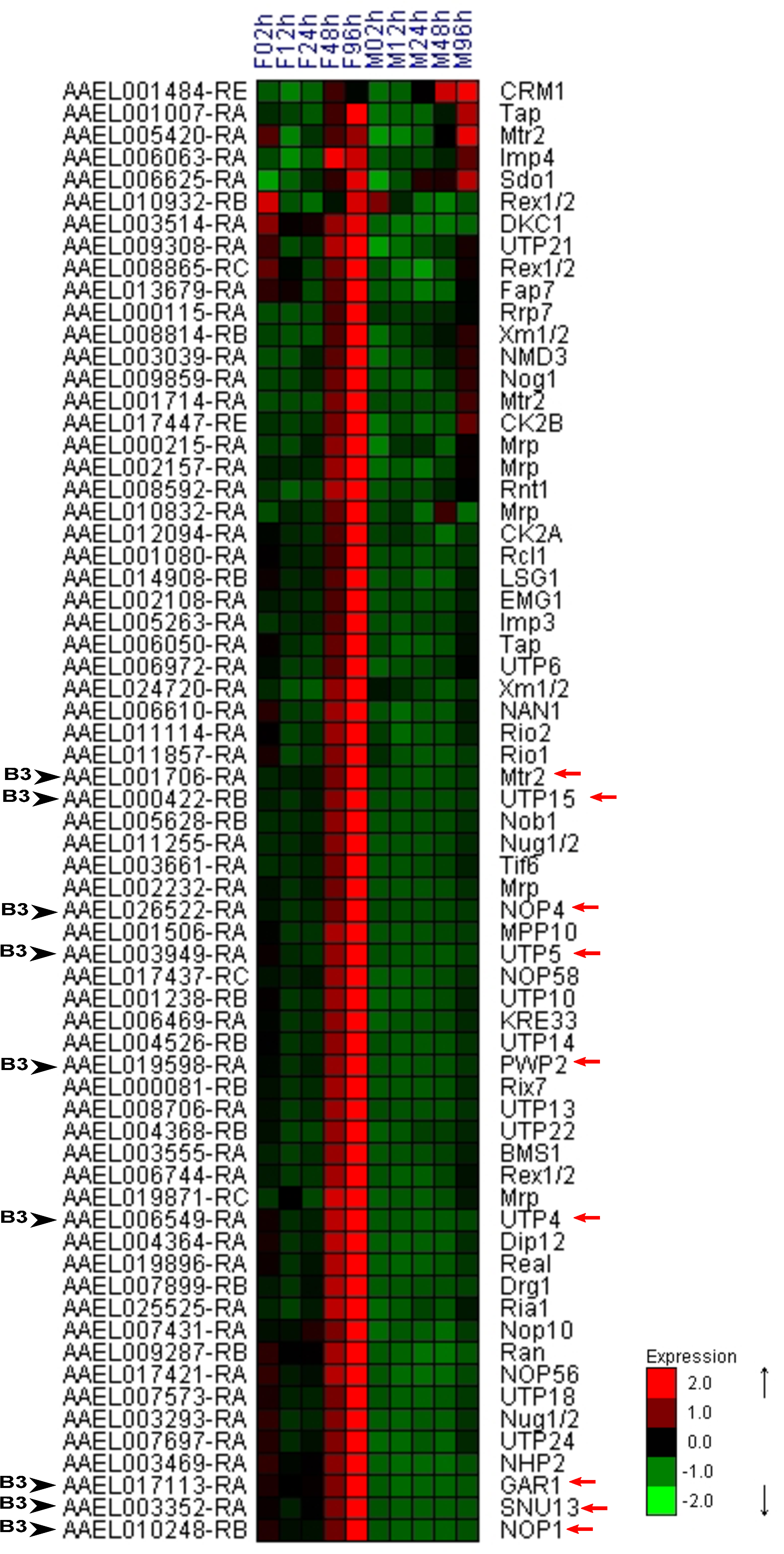

Supplement: S7 Fig — Hierarchical clusterization of the ribosome biogenesis pathway in the post-emergence time points of 2, 12, 24, 48, and 96 hours. Heatmap y-axis shows gene codes and x-axis the female (F) and male (M) time courses from 2 to 96 hours. B3 is a cluster name described in Fig 1. Up-regulated genes are highlighted by red arrows for females. (TIF) [file pntd.0008915.s007.tif]

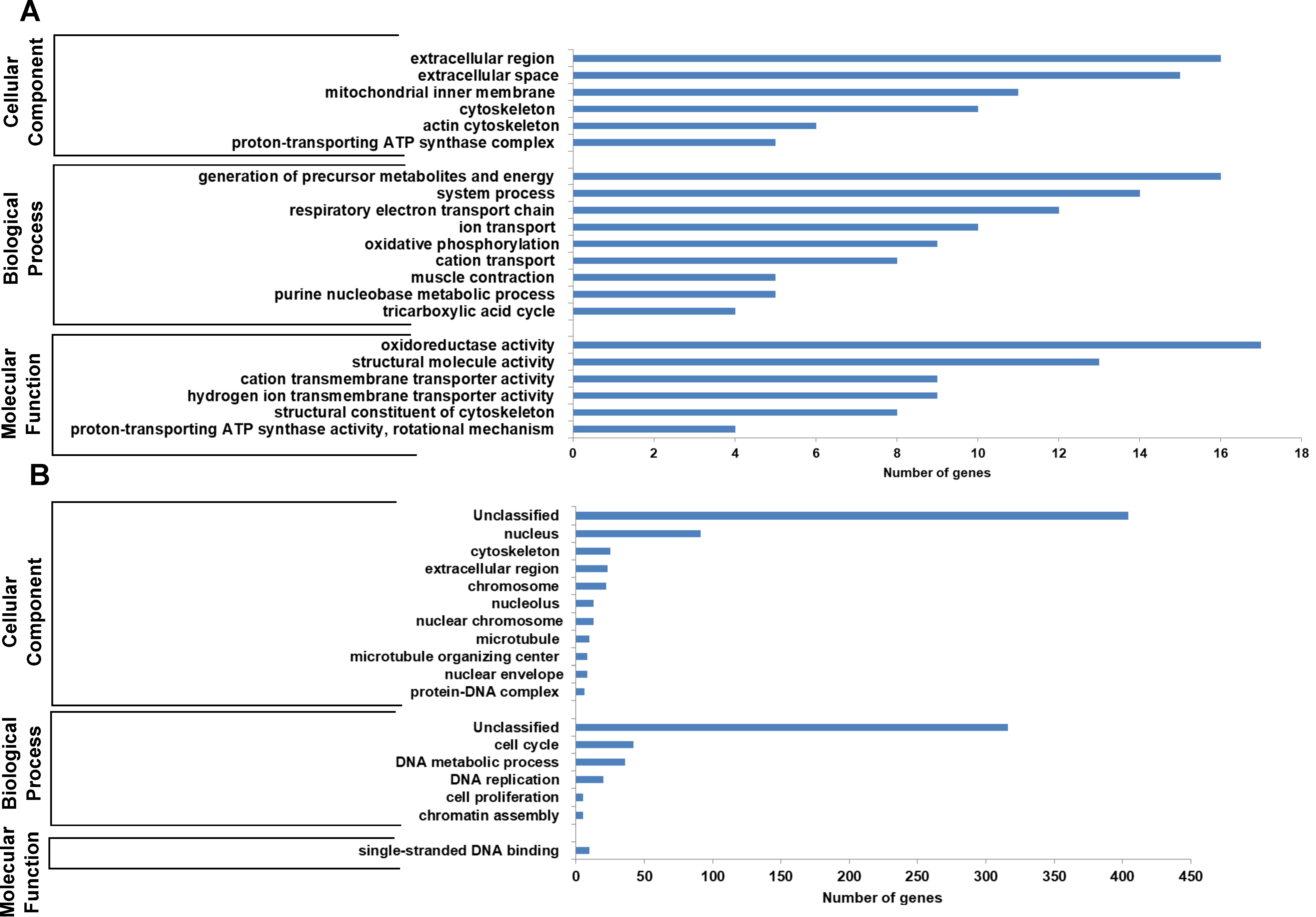

Supplement: S8 Fig — Enrichment analysis was performed with Panther scoring tool. The y-axis shows the GOs enriched for each ontology, as the x-axis the number of genes for the body (A) and the head (B). (TIF) [file pntd.0008915.s008.tif]

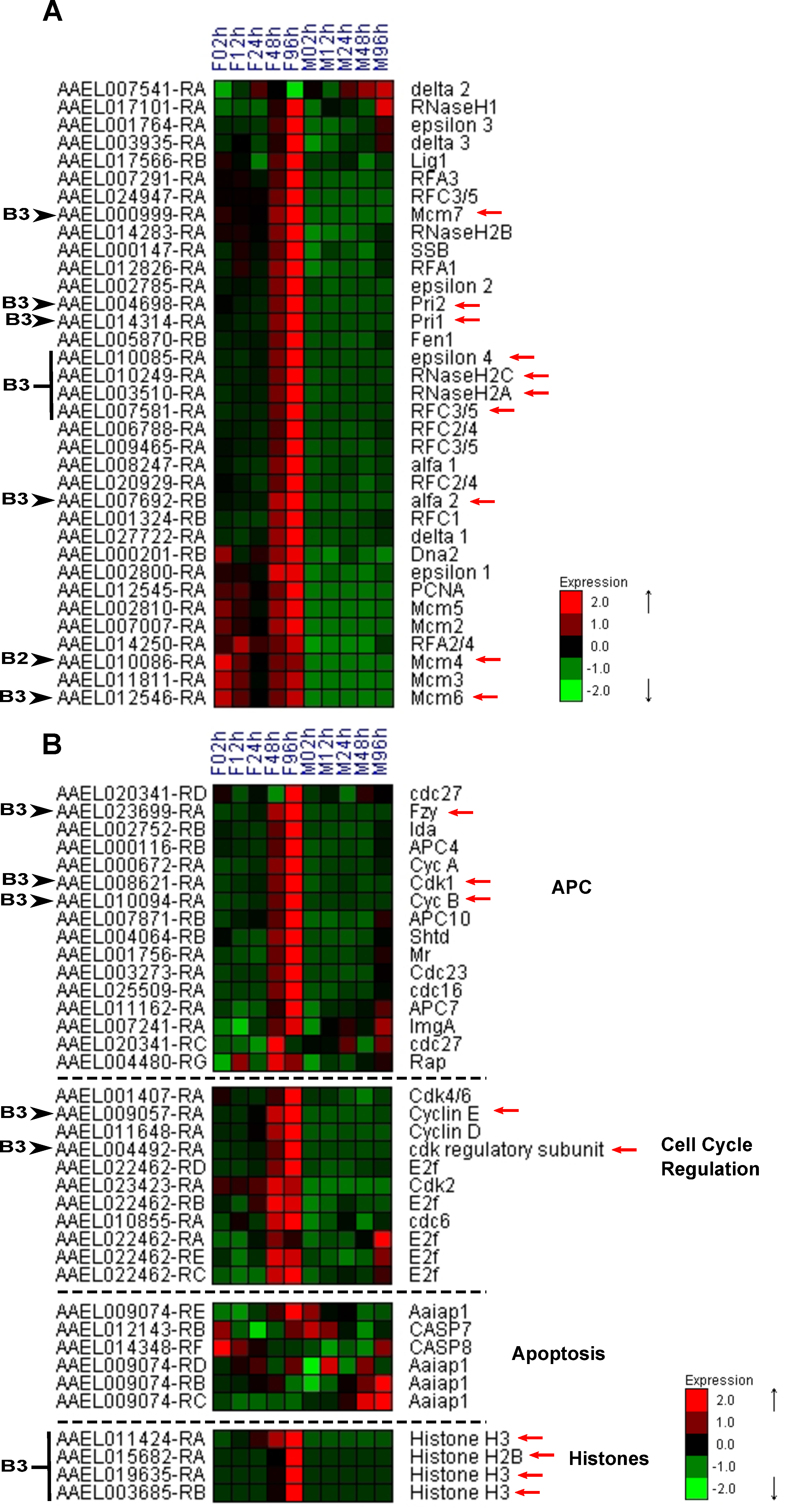

Supplement: S9 Fig — Hierarchical clusterization heatmap for the DNA replication pathway (A) and body differentially expressed genes (DEGs) for cell cycle progression, apoptosis and histones (B). The y-axis shows gene codes and x-axis shows female (F) and male (M) time courses from 2 to 96 hours. B2 and B3 are cluster names described in Fig 1. Up-regulated genes are highlighted by red arrows for females. (TIF) [file pntd.0008915.s009.tif]

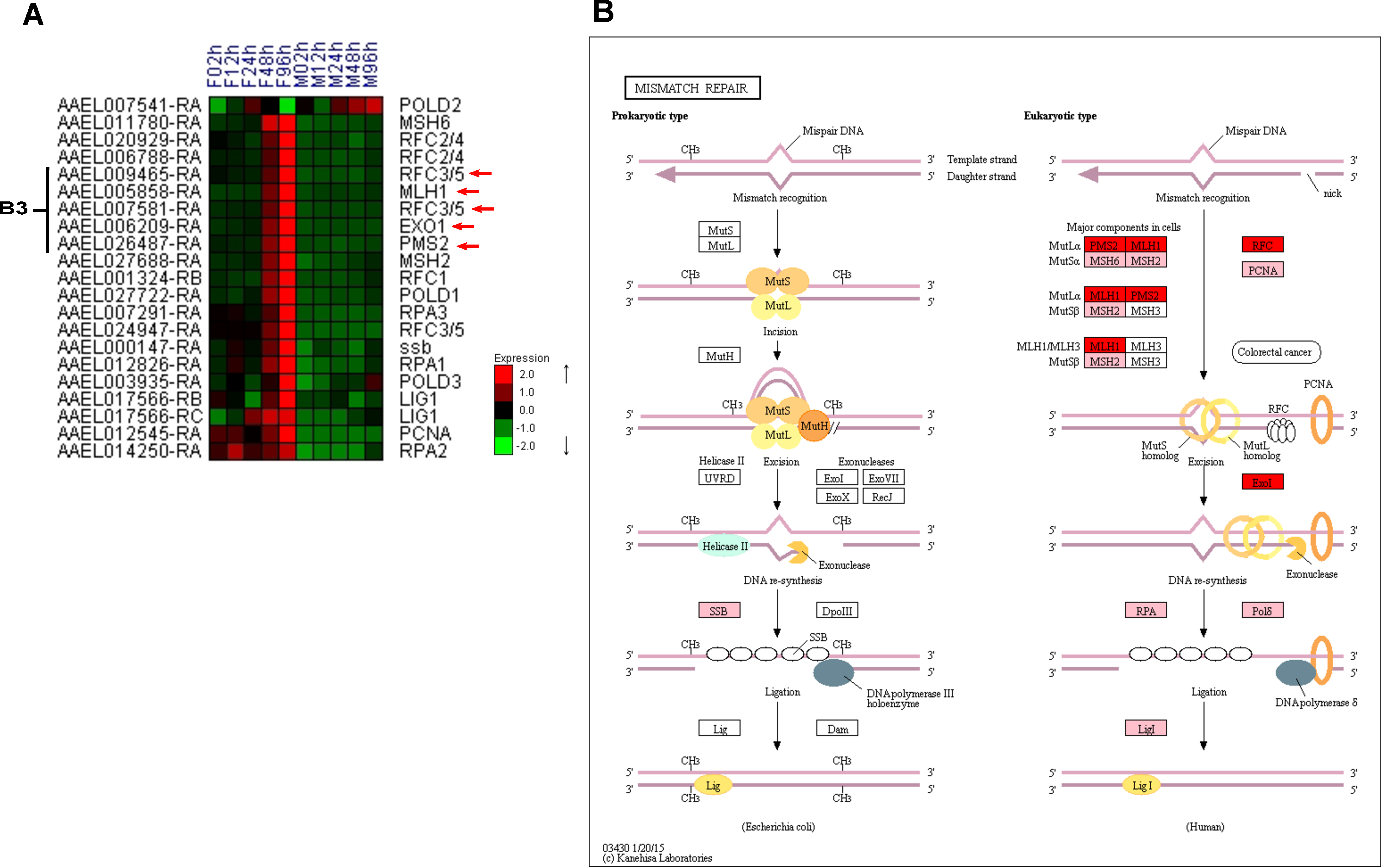

Supplement: S10 Fig — The hierarchical clusterization heatmap y-axis shows gene codes and x-axis shows female (F) and male (M) time courses from 2 to 96 hours. B3 is a cluster name described in Fig 1. Up-regulated genes are highlighted by red arrows for females (A). Differentially expressed genes (DEG) names are in red boxes (up-regulated in female body—group B3), genes not DEG but with similar expression profile in pink, dissimilar or invariable in green and absent genes in white (Source: KEGG Mapper tool) (B). (TIF) [file pntd.0008915.s010.tif]

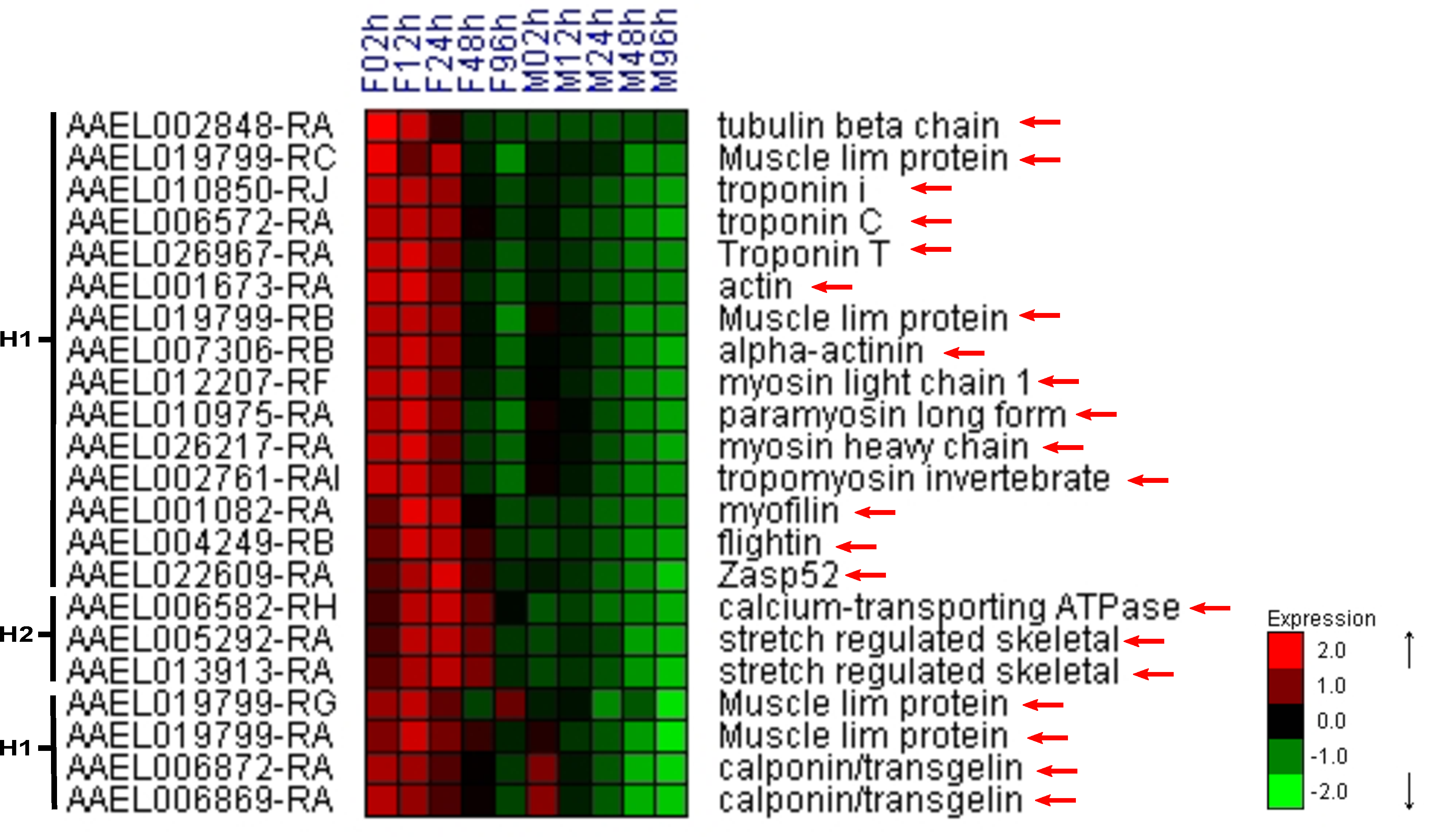

Supplement: S11 Fig — The hierarchical clusterization heatmap y-axis shows gene codes and x-axis shows female (F) and male (M) time courses from 2 to 96 hours. H1 and H2 are cluster names described in Fig 1. Up-regulated genes are highlighted by red arrows for females. (TIF) [file pntd.0008915.s011.tif]

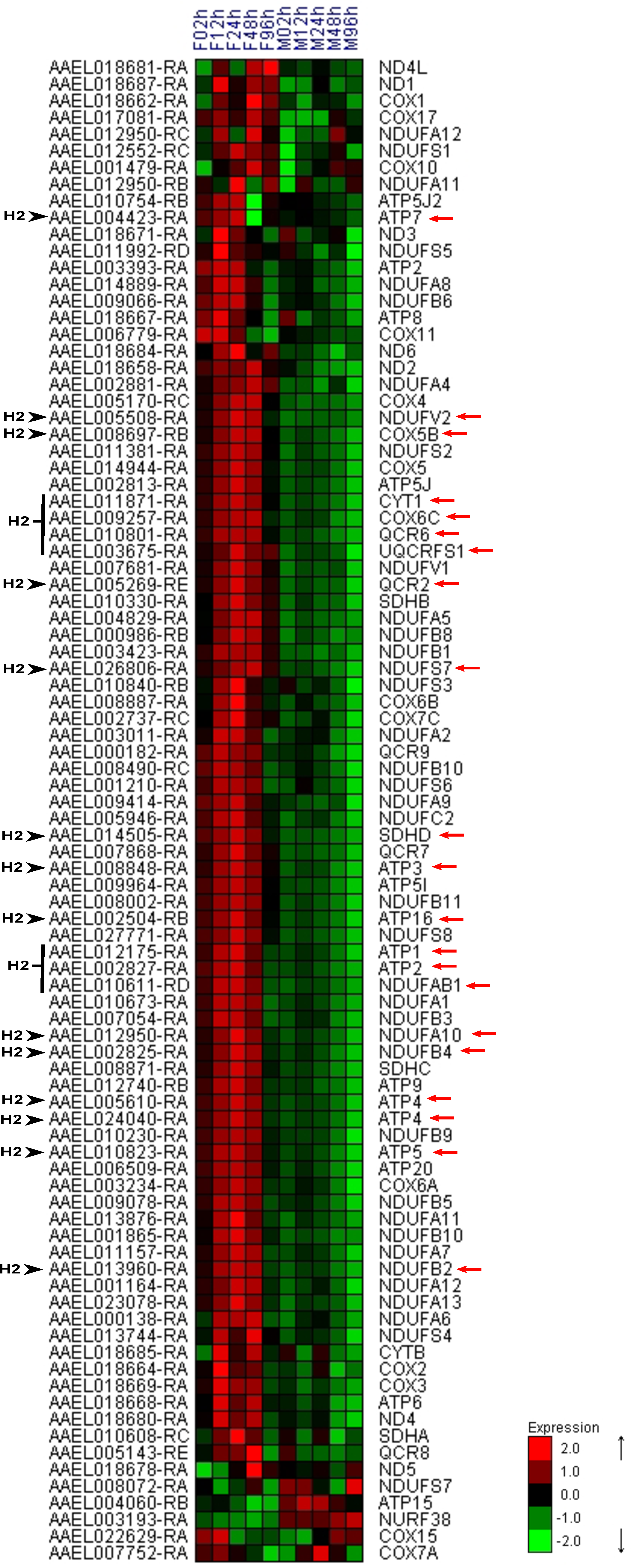

Supplement: S12 Fig — The hierarchical clusterization heatmap y-axis shows gene codes and x-axis shows female (F) and male (M) time courses from 2 to 96 hours. H2 is a cluster name described in Fig 1. Up-regulated genes are highlighted by red arrows for females. (TIF) [file pntd.0008915.s012.tif]

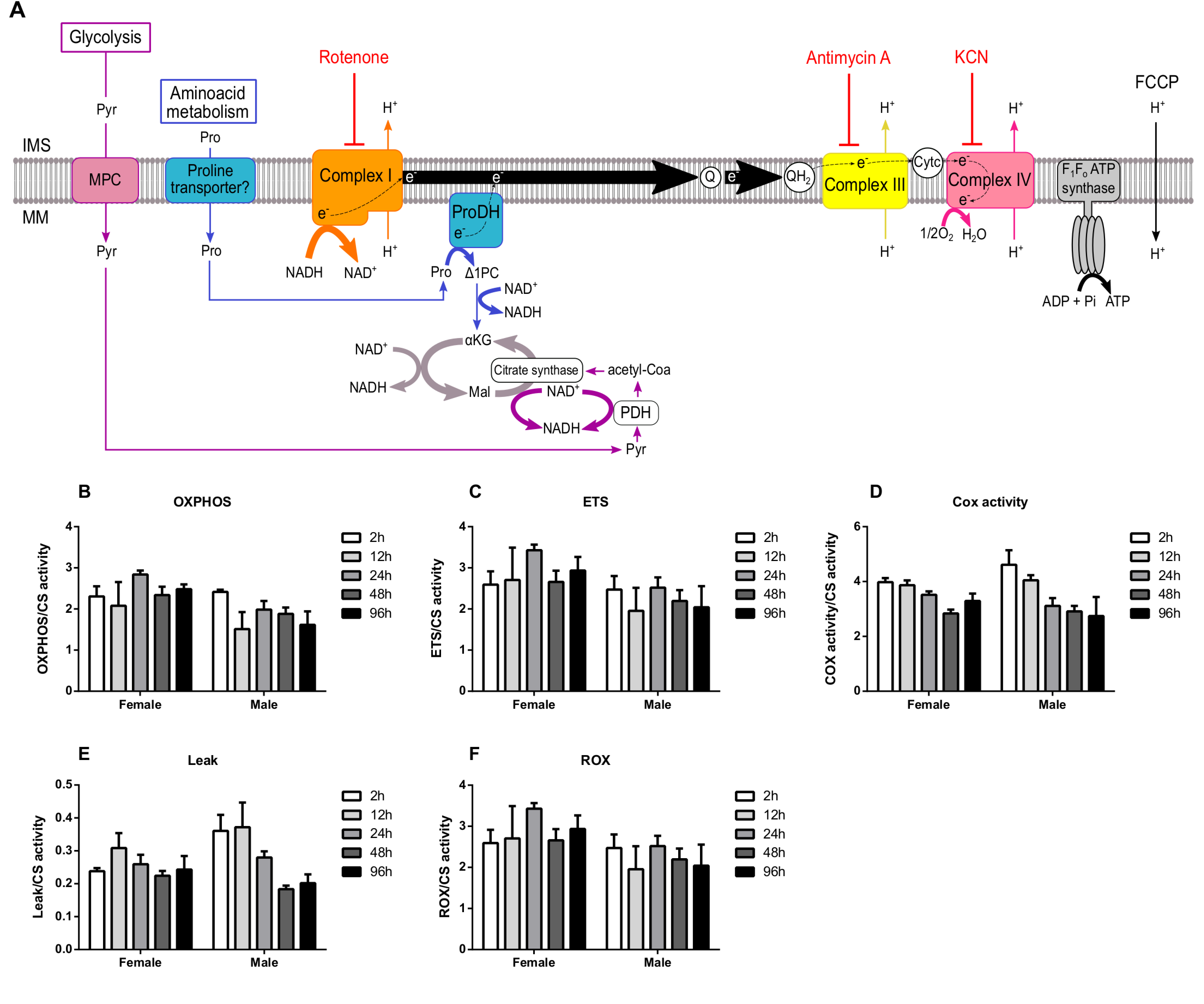

Supplement: S13 Fig — The mitochondrial pyruvate carrier (MPC 1 and MPC2) transports the pyruvate across the mitochondrial inner membrane into the mitochondrial matrix. The pyruvate dehydrogenase complex is composed by pyruvate dehydrogenase A (PDHA) and B (PDHB), dihydrolipoyllysine-residue acetyltransferase oxydase (DLAT) and dihydrolipoyl dehydrogenase (DLD). This complex mediates de oxidative decarboxylation of pyruvate to acetyl-CoA, producing CO2 and NADH. Proline is a main energy source for ATP synthesis in insects through OXPHOS. Although the molecular nature of mitochondrial proline transporter remains unknown, the first step of proline metabolism is mediated by proline desidrogenase (ProDH), generating 1-pyrroline-5-carboxylate (Δ1PC) and ubiquinol. Δ1PC is then oxidised to a-ketoglutarate (a-KG), producing NADH and glutamate. The expression profile of the MPCs and the pyruvate dehydrogenase complex can be observed in Fig 5A. Blunted arrows indicate the inhibitors used for each complex in our experiments (A). The head OCRs were normalized by CS activity. Oxygen consumption coupled with oxidative phosphorylation (OXPHOS) (B); maximum respiratory rates (ETS) (C); Cytochrome c oxidase activity (D); Leak represents the oxygen consumption in the presence of high substrate concentration but in the absence of ADP (E); residual oxygen consumption (ROX) (F). Bar graphs show mean (SEM) for males and females at 2, 12, 24, 48, and 96h post-emergence. Interaction p-value between sex and time factors (two-way ANOVA) were not significant (p>0.05) for all panels. (TIF) [file pntd.0008915.s013.tif]

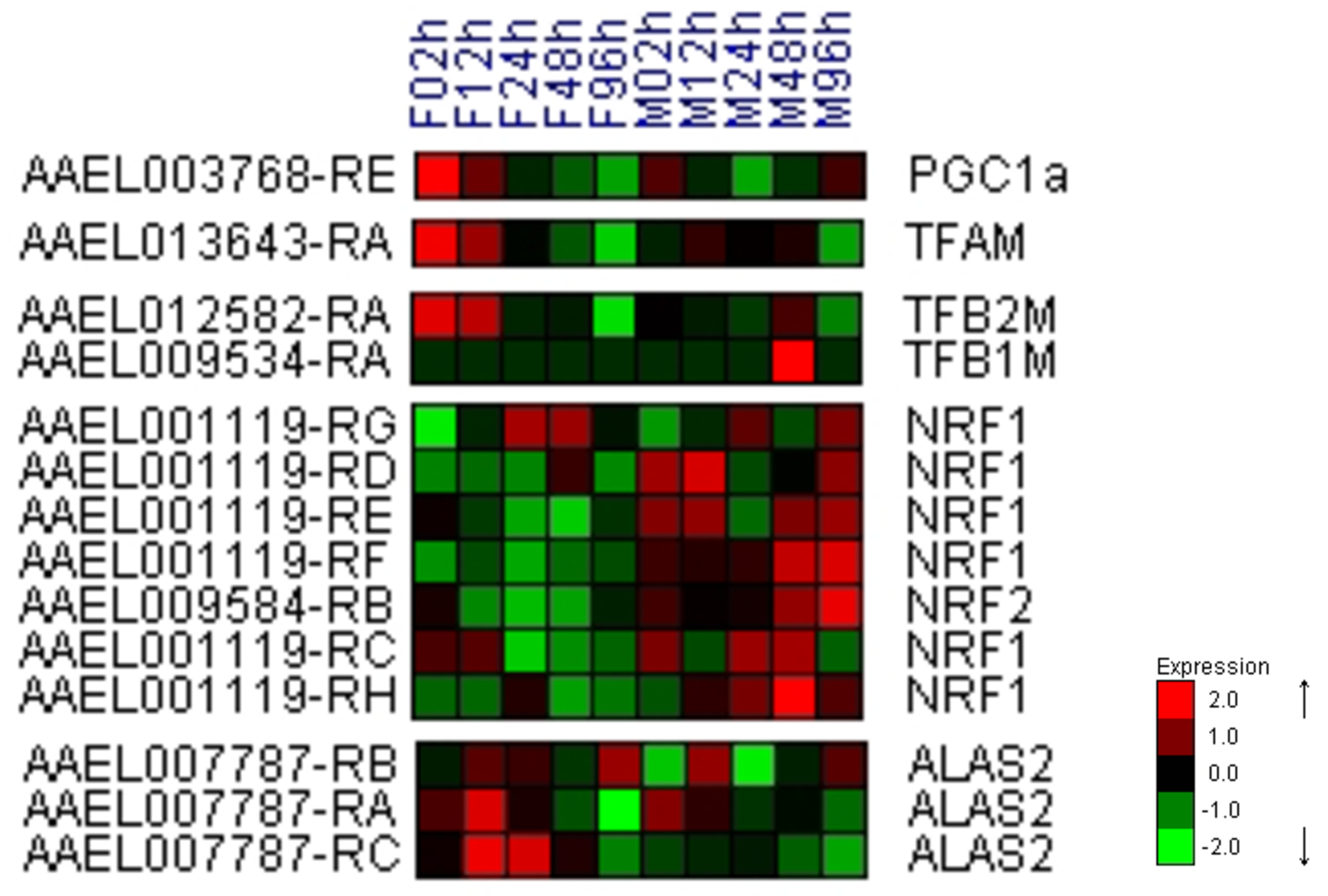

Supplement: S14 Fig — The hierarchical clusterization heatmap y-axis shows gene codes and x-axis shows female (F) and male (M) time courses from 2 to 96 hours. (TIF) [file pntd.0008915.s014.tif]
